# Changes in notifiable infectious disease incidence in China during the COVID-19 pandemic

**DOI:** 10.1038/s41467-021-27292-7

**Published:** 2021-11-26

**Authors:** Meng-Jie Geng, Hai-Yang Zhang, Lin-Jie Yu, Chen-Long Lv, Tao Wang, Tian-Le Che, Qiang Xu, Bao-Gui Jiang, Jin-Jin Chen, Simon I. Hay, Zhong-Jie Li, George F. Gao, Li-Ping Wang, Yang Yang, Li-Qun Fang, Wei Liu

**Affiliations:** 1 Division of Infectious Disease, Key Laboratory of Surveillance and Early-warning on Infectious Disease, Chinese Center for Disease Control and Prevention, Beijing, China; 2 State Key Laboratory of Pathogen and Biosecurity, Beijing Institute of Microbiology and Epidemiology, Beijing, P. R. China; 3 Center for Disease Control and Prevention of Central Theater Command, Shijingshan District, Beijing, China; 4 Department of Health Metrics Sciences, School of Medicine, University of Washington, Seattle, WA USA; 5 Institute for Health Metrics and Evaluation, University of Washington, Seattle, WA USA; 6 Department of Biostatistics, College of Public Health and Health Professions, and Emerging Pathogens Institute, University of Florida, Gainesville, FL USA

**Keywords:** Viral infection, Epidemiology, Bacterial infection

## Abstract

Nationwide nonpharmaceutical interventions (NPIs) have been effective at mitigating the spread of the novel coronavirus disease (COVID-19), but their broad impact on other diseases remains under-investigated. Here we report an ecological analysis comparing the incidence of 31 major notifiable infectious diseases in China in 2020 to the average level during 2014-2019, controlling for temporal phases defined by NPI intensity levels. Respiratory diseases and gastrointestinal or enteroviral diseases declined more than sexually transmitted or bloodborne diseases and vector-borne or zoonotic diseases. Early pandemic phases with more stringent NPIs were associated with greater reductions in disease incidence. Non-respiratory diseases, such as hand, foot and mouth disease, rebounded substantially towards the end of the year 2020 as the NPIs were relaxed. Statistical modeling analyses confirm that strong NPIs were associated with a broad mitigation effect on communicable diseases, but resurgence of non-respiratory diseases should be expected when the NPIs, especially restrictions of human movement and gathering, become less stringent.

## Abstract (web summary)

Non-pharmaceutical interventions implemented to mitigate COVID-19 transmission are likely to have impacted spread of other infectious diseases. Here, the authors investigate changes in the incidence of 31 notifiable infectious diseases using surveillance data from China.

## Introduction

In response to the ongoing pandemic of the coronavirus disease 2019 (COVID-19), a variety of public health interventions have been implemented worldwide to mitigate the impact of the pandemic, including both nonpharmaceutical (social distancing, mask-wearing, shelter-in-place, travel restrictions, school closure, etc.) and pharmaceutical measures (ventilator, antibodies, vaccines, etc.). While pharmaceutical interventions generally target specific pathogens, nonpharmaceutical measures can act on a broad spectrum of infectious pathogens. For example, school closure has been shown to mitigate the spread of seasonal influenza viruses^1–5^. Case identification and quarantine of close contacts via contact tracing have successfully curtailed newly emerging pathogens such as Ebola virus before vaccines became available^6^. To plan preventive programs for emerging and re-emerging diseases in the post-pandemic era, it is necessary to understand the broad impact of the nonpharmaceutical interventions on common endemic infectious diseases other than COVID-19 during the pandemic.

Several studies have explored the impact of the nonpharmaceutical interventions during the COVID-19 pandemic on other pathogens^7–12^. However, most studies focused solely on respiratory pathogens such as influenza virus, respiratory syncytial virus (RSV), and Mycobacterium tuberculosis. Although respiratory pathogens were likely affected the most by the nonpharmaceutical interventions, pathogens with other transmission modes, e.g., gastrointestinal, sexually transmitted, or even vector-borne diseases, could have also been affected as the unprecedented changes in human movement and behavioral patterns might have changed exposure levels. For instance, incidence of norovirus was dramatically decreased in nine states in the United States due to NPIs^13^. In China, where COVID-19 was first reported, the trends of common infectious diseases during and after the first epidemic wave have not been systematically investigated at the national level, although studies on a particular disease or in a specific region have been reported^14,15^.

In this study, we aim to quantify the changes in the incidences of notifiable infectious diseases with different transmission modes before, during, and after the first wave of COVID-19 in the mainland of China and to assess how these changes relate to the public health interventions, using the national surveillance data from 2014 to 2020.

## Results

### Overall trend by disease category

Among the 40 notifiable infectious diseases in mainland China, we selected 31 for this study. During 2014–2020, these diseases were diagnosed in over 53 million individuals with a median age of 17 (IQR: 2–46) years, 59.83% of whom were male. The data cover all 31 provinces (Supplementary Fig. 1). The proportions of laboratory confirmations among all reported cases were relatively stable for all the diseases throughout the study period (Supplementary Fig. 2), and the proportions were comparable across all provinces (Supplementary Fig. 3), suggesting that laboratory-based diagnosis of notifiable disease was not qualitatively affected by diversion of laboratory resources to the diagnosis of COVID-19 during the pandemic. The highest average annual incidence during 2014–2019 was seen for gastrointestinal or enteroviral diseases, followed by respiratory diseases, sexually transmitted or blood-borne diseases, and vector-borne or zoonotic diseases (Table 1). There was a clear age difference, e.g., children and adolescents had higher incidences of respiratory diseases and gastrointestinal or enteroviral diseases than adults, while the opposite is true for sexually transmitted or bloodborne diseases and vector-borne or zoonotic diseases (Supplementary Fig. 4). Differences between sexes or across regions were relatively small, although gastrointestinal or enteroviral disease incidence was slightly higher in males and in southern China.

**Table 1:** Comparison of crude average annual incidence (/100 000) of 31 notifiable infectious diseases between 2014–2019 and 2020 in the mainland of China.

Disease	Overall		Phase I^†^		Phase II^†^		Phase III^†^		Phase IV^†^	
	2014‒2019	2020	2014‒2019	2020	2014‒2019	2020	2014‒2019	2020	2014‒2019	2020
*Respiratory diseases*	156.24	145.12	15.44	62.99	36.07	25.45	51.89	30.51	52.84	26.17
Seasonal influenza	66.69	82.29	8.99	57.81	17.98	13.49	13.94	4.96	25.79	6.04
Tuberculosis	64.27	51.42	5.06	3.70	14.18	10.29	25.70	21.78	19.33	15.65
Mumps	16.83	9.60	0.93	0.94	2.40	1.39	8.18	3.42	5.32	3.85
Scarlet fever	5.12	1.25	0.34	0.40	0.63	0.11	2.23	0.24	1.91	0.49
Measles	1.59	0.06	0.07	0.01	0.54	0.01	0.85	0.02	0.13	0.02
Pertussis	0.97	0.34	0.03	0.06	0.16	0.14	0.52	0.06	0.25	0.08
Rubella	0.76	0.16	0.02	0.07	0.17	0.03	0.47	0.02	0.10	0.03
*Gastrointestinal or enteroviral diseases*	261.92	142.16	10.53	8.72	29.44	11.62	143.41	46.19	78.56	75.64
Hand, foot and mouth disease	164.71	56.42	3.56	1.66	11.09	0.71	103.33	7.45	46.74	46.60
Infectious diarrhea	81.49	76.36	6.30	6.57	15.92	9.54	32.22	34.19	27.04	26.07
Bacterial dysentery	8.43	4.19	0.27	0.17	1.04	0.47	4.68	2.29	2.44	1.25
Acute hemorrhagic conjunctivitis	2.79	2.16	0.12	0.12	0.43	0.35	1.31	0.96	0.94	0.73
Hepatitis E	2.04	1.37	0.15	0.10	0.51	0.24	0.79	0.58	0.59	0.45
Hepatitis A	1.51	1.08	0.09	0.07	0.31	0.23	0.61	0.43	0.50	0.34
Typhoid and paratyphoid	0.87	0.56	0.04	0.03	0.13	0.08	0.43	0.27	0.28	0.18
Amebic dysentery	0.08	0.05	0.004	0.003	0.01	0.01	0.04	0.02	0.02	0.02
*Sexually transmitted or bloodborne diseases*	148.48	137.08	10.15	10.27	30.30	22.09	60.77	58.57	47.25	46.14
Hepatitis B	75.19	67.57	5.64	5.52	16.23	11.43	30.23	28.60	23.08	22.03
Syphilis	35.11	34.95	2.08	2.35	6.69	5.56	14.78	15.21	11.56	11.83
Hepatitis C	15.85	13.94	1.14	1.05	3.36	2.23	6.42	5.95	4.94	4.70
HIV/AIDS	13.74	12.97	0.75	0.87	2.55	2.09	5.80	5.61	4.65	4.41
Gonorrhea	8.58	7.64	0.53	0.49	1.48	0.78	3.54	3.20	3.03	3.16
*Vector-borne or zoonotic diseases*	6.64	4.48	0.30	0.23	1.09	0.71	2.79	2.17	2.46	1.39
Dengue	1.08	0.06	0.002	0.004	0.01	0.003	0.16	0.01	0.91	0.04
Schistosomiasis	0.56	0.003	0.01	0.0001	0.09	0.0005	0.19	0.001	0.27	0.001
Malaria	0.22	0.08	0.01	0.02	0.04	0.02	0.10	0.02	0.06	0.02
Typhus	0.09	0.09	0.0032	0.003	0.01	0.01	0.04	0.04	0.04	0.03
Japanese encephalitis	0.08	0.02	0.00006	0	0.0003	0.00007	0.06	0.01	0.02	0.01
Brucellosis	3.41	3.36	0.19	0.16	0.73	0.57	1.79	1.77	0.70	0.87
Hemorrhagic fever with renal syndrome	0.78	0.59	0.05	0.04	0.13	0.07	0.27	0.19	0.32	0.29
Hydatid diseases.	0.32	0.24	0.03	0.02	0.07	0.03	0.12	0.10	0.11	0.09
Rabies	0.04	0.02	0.003	0.001	0.01	0.002	0.02	0.01	0.02	0.01
Anthrax	0.03	0.02	0.0007	0.0009	0.003	0.002	0.02	0.01	0.01	0.01
Leptospirosis	0.02	0.03	0.0002	0.0002	0.0009	0.0003	0.01	0.01	0.01	0.02

All the incidences in the table were crude incidence rate without age adjustment.

The denominator to calculate the incidence of 2014‒2019 was 1383.7 million and the denominator to calculate the incidence of 2020 was 1403.9 million.

^†^Phase I: Jan 1 to Jan 22; Phase II: Jan 23 to Apr 7; Phase III: Apr 8 to Aug 31; Phase IV: Sep 1 to Dec 31.

The annual incidence of the 31 infectious diseases combined decreased from 573.28 per 100,000 people during 2014–2019 to 428.88 in 2020, with the largest drop in the gastrointestinal or enteroviral diseases from 261.92 to 142.16 per 100,000, a 45.7% reduction, followed by the vector-borne or zoonotic diseases, a 32.5% reduction (Table 1). In Phase I (Jan 1–Jan 22) when NPI had not been massively initiated, a 308% jump was seen in the overall respiratory disease incidence. Comparing the phases with massive NPIs in 2020 to the corresponding time intervals of 2014–2019, gastrointestinal or enteroviral diseases had the largest decreases in both Phase II from Jan 23 to Apr 7 (60.5%) and Phase III during Apr 8–Aug 31 (67.8%), followed by vector-borne or zoonotic diseases in Phase II (34.8%) and respiratory diseases in Phase III (41.2%). In Phase IV, respiratory diseases took the lead with a 50.5% reduction, followed by vector-borne or zoonotic diseases (43.5%).

The overall case-fatality ratio (CFR), however, increased from 4.34 per 1000 cases during 2014–2019 to 6.70 per 1000 cases in 2020 (Supplementary Table 1), mainly driven by the increase in sexually transmitted or blood-borne disease-associated deaths (19.68 in 2020 vs. 14.89 in 2014‒2019 per 1000 cases). In contrast, the CFRs of the respiratory diseases, gastrointestinal or enteroviral diseases, and vector-borne or zoonotic diseases were significantly reduced in 2020 compared with 2014–2019. The CFRs of seasonal influenza, tuberculosis, and HFMD declined, while those of typhoid or paratyphoid, hepatitis B, HIV/AIDS, and leptospirosis increased significantly.

### Trend at the province level by disease category

**Fig. 1:**
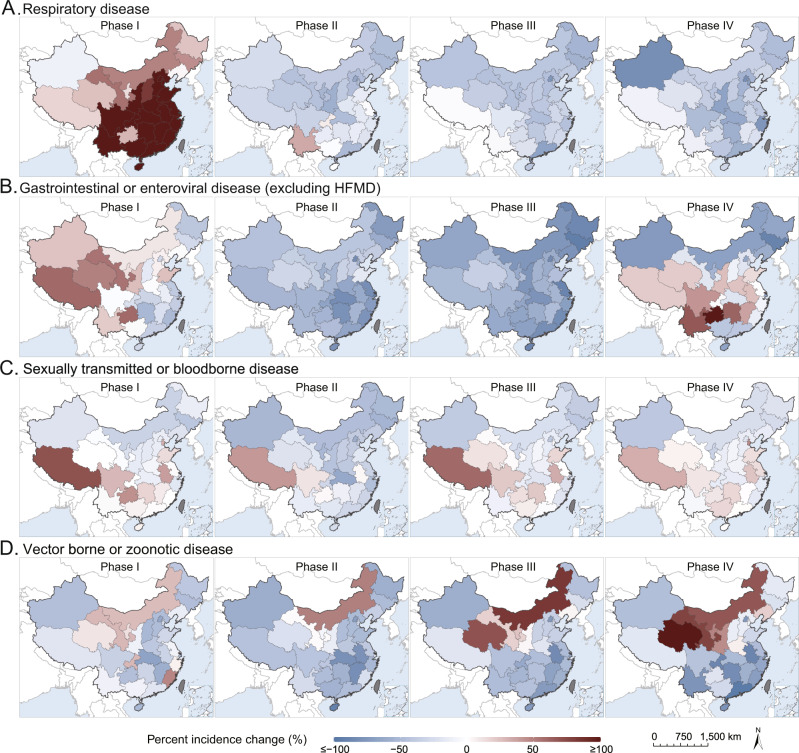
Percent changes of cumulative incidence in each epidemic phase between 2020 and the average of 2014−2019 at the province level for four categories of notifiable infectious diseases. **A** Respiratory disease, **B** gastrointestinal or enteroviral disease (excluding HFMD), **C** sexually transmitted or blood-borne disease, **D** vector-borne or zoonotic disease. The epidemic phases are defined as before Jan 22 (Phase I), Jan 23–Apr 7 (Phase II), Apr 8–Aug 31 (Phase III) and after Sep 1 (Phase IV). Gradient red and blue colors indicate positive and negative changes from 2014 to 2019, shaded gray indicates the missing value.

Comparing 2020 to 2014–2019, increases of the overall respiratory-disease incidence in Phase I and decreases in subsequent phases were observed in almost all 31 provinces, although the increases in Phase I tended to be more intense in more densely populated provinces in southern and eastern China (Fig. 1A). Changes in the provincial overall gastrointestinal or enteroviral disease incidence (excluding HFMD) showed more spatial heterogeneity, featuring decreases in the east but increases in the west during phase I, uniform decreases in the whole nation during phases II and III, and large rebounds in the central and western provinces in Phase IV (Fig. 1B). Change patterns of the provincial overall sexually transmitted or blood-borne disease incidence and vector-borne or zoonotic disease incidence were similar across phases (Fig. 1C, D). Increases in the sexually transmitted or bloodborne diseases overall incidence from 2014–2019 to 2020 occurred in Tibet and several central and eastern provinces (Fig. 1C), whereas increases in the vector-borne or zoonotic disease incidence were observed in northern provinces, particularly Inner Mongolia where vector-borne diseases have been endemic (Fig. 1D). In Phase II when the NPIs were most intense, many provinces with increased sexually transmitted or blood-borne diseases and vector-borne or zoonotic disease incidences in other phases showed decreases. Densely populated provinces in the east and the south also showed persistent decreases in vector-borne or zoonotic disease incidence throughout phases II–IV.

In Phase I, the nationwide annual incidence of seasonal influenza rose sharply from 8.99 during 2014–2019 to 57.81 per 100,000 in 2020, which drove the increase of the overall respiratory-disease incidence (Table 1). Small increases were also seen for scarlet fever, pertussis, and rubella in Phase I. In contrast, disease-specific incidences all decreased during phases II‒IV in 2020. The largest decreases were associated with seasonal influenza during Phase III (13.94 vs. 4.96 per 100,000) and Phase IV (25.79 vs. 6.04 per 100,000) (Table 1, Supplementary Fig. 5). Disease-specific change patterns were spatially heterogeneous at the provincial level, but the general pattern was similar to the national trend of increase in Phase I and decreases in later phases (Supplementary Fig. 6). The only exceptions are measles and TB. Measles incidence was decreasing in most provinces even in Phase I. TB was the least impacted by COVID-19 among these respiratory diseases, likely due to its chronic nature. All age groups showed increasing seasonal influenza and pertussis incidences in Phase I and decreasing incidences for nearly all respiratory diseases in phases II–IV, except that mumps rebounded among children <5 years old in Phase IV (Supplementary Fig. 7). No sex difference was found in the respiratory-disease incidence changes (Supplementary Fig. 7).

As the predominant driver for the overall gastrointestinal or enteroviral disease incidence, the annual incidence of HFMD plunged 66% from 164.71 during 2014–2019 to 56.42 per 100,000 in 2020, with the largest drops in Phases II (93.6%) and III (92.8%) (Table 1, Supplementary Fig. 5). However, HFMD rebounded to its historical level in Phase IV. Infectious diarrhea caused by unidentified pathogens decreased only briefly in Phase III, while other gastrointestinal or enteroviral diseases declined in nearly all phases (Supplementary Fig. 5). Both HFMD and infectious diarrhea were increasing in many western and northern provinces during Phase I of 2020, and both had a rebound in southwestern and central regions during Phase IV (Supplementary Fig. 8). Acute hemorrhagic conjunctivitis rebounded in northern and northeastern provinces in Phase IV. Of note, Tibet had increasing hepatitis E in most phases. Children <5 years old were the only age group with decreased infectious diarrhea in most phases (Supplementary Fig. 9A–D). The gastrointestinal or enteroviral disease incidence changes were similar between males and females (Supplementary Fig. 9E).

Like the overall sexually transmitted or blood-borne disease incidence, the incidences of specific sexually transmitted or bloodborne diseases decreased noticeably only in Phase II, with gonorrhea, hepatitis C and hepatitis B being the top contributors associated with declines of 47.3%, 33.6%, and 29.6%, respectively (Table 1 and Supplementary Fig. 5). The incidence change patterns of hepatitis B, syphilis, and gonorrhea recovered in Phase III to comparable levels in Phase I in many provinces, especially those in southwestern China (Supplementary Fig. 10). Gonorrhea remained increasing in the southwest in most phases. Children aged <5 years are the only group with persistent decreases in these sexually transmitted or blood-borne diseases throughout all phases (Supplementary Fig. 11). Surprisingly, the increasing trend of syphilis and gonorrhea remained throughout all phases among the adolescents 5–17 years old. The decline of HIV/AIDS in Phase II among the elderly was the modest of all the five sexually transmitted or blood-borne diseases, and the incidence resumed its increasing trend in Phase III. No sex difference was found in the change patterns of the sexually transmitted or blood-borne diseases.

All the selected vector-borne or zoonotic diseases had reduced incidences in Phases II and III, and this trend carried over to Phase IV, except for brucellosis, which is associated with the highest burden of disease among all vector-borne or zoonotic diseases in the mainland of China^16^, and leptospirosis (Table 1). Dengue and malaria showed about 100% increase in Phase I, and the largest incidence drop in Phase IV was seen in dengue, which used to peak in fall in the south via both importation and local outbreaks (Supplementary Fig. 5). Brucellosis showed persistent incidence increases throughout all phases in western and northern provinces, except for Xinjiang, and rebounds in phases III and IV were seen in eastern and southeastern China (Supplementary Fig. 12). All age groups shared similar change patterns of vector-borne or zoonotic diseases, except that malaria rebounded quickly in Phase IV only among children <5 years old (Supplementary Fig. 13). Schistosomiasis incidence dropped in most age groups across all phases (Supplementary Fig. 13).

We selected nine diseases to perform a sensitivity analysis based on only lab-confirmed cases. For most of these diseases, the phase-specific change patterns from 2014–2019 to 2020 among lab-confirmed cases were similar to those of all confirmed cases, featuring magnificent declines in phases II and III together with either a mild decline (most diseases) or a rebound (HFMD and brucellosis) in Phase IV (Supplementary Fig. 14). An increase in the annual incidence of lab-confirmed cases was observed for seasonal influenza in Phase II and for TB in all phases, which might be related to the higher proportions of laboratory diagnoses among all diagnoses of the two diseases in 2020 compared with the previous years (Supplementary Fig. 2).

### Model-estimated association of NPIs with disease trend

After excluding diseases with a median monthly number of reported cases <50 during 2014–2020, 26 notifiable infectious diseases were included in generalized linear model analysis (Table 2). Healthcare-seeking behavior was substantially altered during the COVID-19 pandemic. Compared with 2019, the monthly volume of outpatient visits in 2020 started declining notably in February when intensive NPIs were initiated but gradually recovered to the same level as the previous year by August or September (Supplementary Fig. 15). The magnitude of relative changes and the time to full recovery are comparable across disease categories. For sexually transmitted or blood-borne diseases, the temporal trends are similar between reported cases and outpatient visits. For all other disease categories, the temporal variation of reported cases cannot be explained by that of outpatient visits. To tease out a more objective association of NPIs with disease incidence, the monthly volume of outpatient visits was adjusted for in all models.

**Table 2:** Model-estimated incidence rate ratio (IRR) of 26 selected infectious diseases.

Disease^†^	South						North					
	Phase II^‡^		Phase III^‡^		Phase IV^‡^		Phase II^‡^		Phase III^‡^		Phase IV^‡^	
	IRR (95%CI)	*p* value	IRR (95%CI)	*p* value	IRR (95%CI)	*p* value	IRR (95%CI)	*p* value	IRR (95%CI)	*p* value	IRR (95%CI)	*p* value
*Respiratory diseases*												
Measles	0.55 (0.28−1.06)	0.080	0.78 (0.51−1.18)	0.238	0.72 (0.43−1.20)	0.213	**0.02 (0.001−0.44)**	**0.017**	1.85 (0.37−9.30)	0.460	**4.47 (1.2−16.58)**	**0.028**
Mumps	**0.53 (0.39−0.71)**	**<0.001**	**0.38 (0.32−0.44)**	**<0.001**	**0.33 (0.28−0.39)**	**<0.001**	**0.48 (0.35−0.65)**	**<0.001**	**0.34 (0.28−0.41)**	**<0.001**	**0.40 (0.33−0.48)**	**<0.001**
Pertussis	**0.45 (0.23−0.88)**	**0.022**	**0.06 (0.03−0.10)**	**<0.001**	**0.12 (0.08−0.18)**	**<0.001**	**0.23 (0.13−0.40)**	**<0.001**	**0.05 (0.03−0.08)**	**<0.001**	**0.11 (0.07−0.17)**	**<0.001**
Rubella	**0.10 (0.02−0.47)**	**0.005**	**0.02 (0.004−0.08)**	**<0.001**	**0.01 (0.004−0.06)**	**<0.001**	**0.18 (0.06−0.59)**	**0.006**	**0.06 (0.02−0.15)**	**<0.001**	**0.11 (0.06−0.23)**	**<0.001**
Scarlet fever	**0.22 (0.15−0.32)**	**<0.001**	**0.26 (0.21−0.32)**	**<0.001**	**0.26 (0.21−0.31)**	**<0.001**	**0.11 (0.07−0.18)**	**<0.001**	**0.10 (0.07−0.14)**	**<0.001**	**0.13 (0.10−0.17)**	**<0.001**
Seasonal influenza	**0.16 (0.03−0.76)**	**0.024**	**0.06 (0.02−0.15)**	**<0.001**	**0.05 (0.02−0.12)**	**<0.001**	**0.21 (0.10−0.44)**	**<0.001**	**0.15 (0.10−0.23)**	**<0.001**	**0.11 (0.07−0.17)**	**<0.001**
Tuberculosis	**0.87 (0.78−0.97)**	**0.014**	0.97 (0.92−1.03)	0.389	0.96 (0.91−1.02)	0.221	0.85 (0.73−1.00)	0.057	**0.86 (0.79−0.94)**	**<0.001**	**0.83 (0.76−0.90)**	**<0.001**
*Gastrointestinal or enteroviral disease*												
AHC	0.67 (0.38−1.18)	0.172	**0.49 (0.36−0.68)**	**<0.001**	**0.46 (0.33−0.65)**	**<0.001**	0.96 (0.83−1.11)	0.577	1.01 (0.93−1.09)	0.844	**1.18 (1.10−1.27)**	**<0.001**
Bacterial dysentery	**0.71 (0.58−0.85)**	**<0.001**	**0.85 (0.77−0.95)**	**0.004**	**0.84 (0.75−0.93)**	**0.002**	**0.62 (0.50−0.75)**	**<0.001**	**0.76 (0.68−0.84)**	**<0.001**	**0.84 (0.75−0.93)**	**0.001**
Hepatitis A	**0.66 (0.54−0.81)**	**<0.001**	**0.83 (0.75−0.92)**	**<0.001**	0.91 (0.82−1.00)	0.060	0.87 (0.70−1.08)	0.219	**0.69 (0.61−0.79)**	**<0.001**	**0.61 (0.54−0.70)**	**<0.001**
Hepatitis E	**0.70 (0.58−0.84)**	**<0.001**	**0.78 (0.71−0.86)**	**<0.001**	**0.80 (0.73−0.88)**	**<0.001**	**0.70 (0.57−0.86)**	**0.001**	**0.78 (0.70−0.87)**	**<0.001**	**0.80 (0.72−0.89)**	**<0.001**
HFMD	**0.10 (0.02−0.49)**	**0.006**	**0.20 (0.10−0.42)**	**<0.001**	0.77 (0.54−1.10)	0.160	**0.10 (0.03−0.33)**	**<0.001**	**0.13 (0.07−0.25)**	**<0.001**	**0.31 (0.21−0.47)**	**<0.001**
Infectious diarrhea	**0.49 (0.31−0.77)**	**0.003**	0.86 (0.69−1.07)	0.172	0.83 (0.67−1.02)	0.084	**0.52 (0.37−0.72)**	**<0.001**	**0.72 (0.62−0.83)**	**<0.001**	**0.77 (0.68−0.88)**	**<0.001**
Typhoid and paratyphoid	**0.71 (0.54−0.94)**	**0.019**	**0.73 (0.64−0.84)**	**<0.001**	**0.70 (0.60−0.80)**	**<0.001**	0.61 (0.37−1.00)	0.054	**0.68 (0.52−0.89)**	**0.007**	**0.68 (0.52−0.88)**	**0.005**
Amebic dysentery	0.78 (0.56−1.09)	0.156	1.17 (0.99−1.39)	0.073	1.13 (0.96−1.34)	0.153	−	−	−	−	−	−
*Sexually transmitted or bloodborne diseases*												
Gonorrhea	0.80 (0.61−1.03)	0.088	**1.25 (1.11−1.42)**	**<0.001**	**1.30 (1.15−1.46)**	**<0.001**	**0.45 (0.37−0.54)**	**<0.001**	**0.82 (0.75−0.89)**	**<0.001**	0.96 (0.88−1.04)	0.320
Syphilis	**0.72 (0.62−0.82)**	**<0.001**	**0.85 (0.79−0.91)**	**<0.001**	**0.84 (0.79−0.90)**	**<0.001**	**0.69 (0.58−0.81)**	**<0.001**	**0.88 (0.81−0.95)**	**0.002**	**0.86 (0.80−0.92)**	**<0.001**
HIV/AIDS	0.93 (0.78−1.11)	0.438	0.92 (0.84−1.00)	0.063	**0.87 (0.80−0.95)**	**0.003**	0.89 (0.75−1.05)	0.172	0.92 (0.84−1.01)	0.072	0.93 (0.85−1.02)	0.111
Hepatitis B	**0.77 (0.68−0.86)**	**<0.001**	0.97 (0.91−1.03)	0.324	0.96 (0.91−1.02)	0.172	**0.69 (0.58−0.81)**	**<0.001**	**0.90 (0.84−0.98)**	**0.013**	**0.88 (0.81−0.95)**	**0.001**
Hepatitis C	**0.69 (0.62−0.77)**	**<0.001**	0.95 (0.91−1.00)	0.075	0.95 (0.91−1.00)	0.065	**0.62 (0.52−0.74)**	**<0.001**	**0.88 (0.80−0.95)**	**0.003**	**0.87 (0.80−0.94)**	**0.001**
*Vector borne or zoonotic disease*												
Brucellosis	0.92 (0.70−1.22)	0.587	**1.21 (1.05−1.40)**	**0.011**	**1.49 (1.30−1.70)**	**<0.001**	1.03 (0.83−1.29)	0.762	1.12 (1.00−1.25)	0.054	**1.34 (1.21−1.48)**	**<0.001**
HFRS	**0.71 (0.53−0.97)**	**0.032**	**0.83 (0.70−0.97)**	**0.022**	**0.78 (0.66−0.92)**	**0.003**	**0.51 (0.37−0.70)**	**<0.001**	**0.61 (0.52−0.72)**	**<0.001**	**0.77 (0.66−0.90)**	**0.002**
Malaria	**0.51 (0.42−0.64)**	**<0.001**	**0.35 (0.30−0.40)**	**<0.001**	**0.42 (0.37−0.49)**	**<0.001**	**0.62 (0.45−0.85)**	**0.004**	**0.12 (0.08−0.17)**	**<0.001**	**0.22 (0.16−0.30)**	**<0.001**
Hydatid disease	0.76 (0.36−1.58)	0.461	**1.99 (1.38−2.89)**	**<0.001**	**1.90 (1.31−2.77)**	**0.001**	0.68 (0.44−1.05)	0.084	0.98 (0.79−1.20)	0.814	1.20 (0.99−1.45)	0.073
Dengue	0.41 (0.05−3.32)	0.404	**0.01 (0.001−0.22)**	**0.004**	**0.01 (0.0004−0.16)**	**0.002**	−	−	−	−	−	−
Typhus	0.62 (0.38−1.01)	0.056	0.90 (0.70−1.16)	0.408	0.93 (0.73−1.19)	0.563	−	−	−	−	−	−

Generalized linear models (GLM) were used for estimating the IRRs measuring the association of NPIs with disease trends. The models were adjusted for monthly healthcare visits and yearly long-term trend after seasonality was removed by a time series method. IRR < 1 with *P* < 0.05 indicates significant decline in incidence rate in year 2020 compared to year 2014–2019. All *p*-values are two-sided and not adjusted for multiple comparisons.

Statistically significant reductions (IRR < 1) are displayed in bolded font.

^*^Data of Hubei and Tibet (both in South China) were not included in modeling due to unavailable healthcare visits data during the COVID-19 pandemic.

**†** HFMD, hand, foot and mouth disease; AHC, acute hemorrhagic conjunctivitis; HFRS, hemorrhagic fever with renal syndrome.

**‡**Phase II: Feb 2020 and Mar 2020; Phase III: Apr 2020 to Aug 2020; Phase IV: Sep 2020 to Dec 2020. The reference period is Jan 2014 to Jan 2020.

–: GLM was not fitted for disease with median monthly case number is less than 50.

**Fig. 2:**
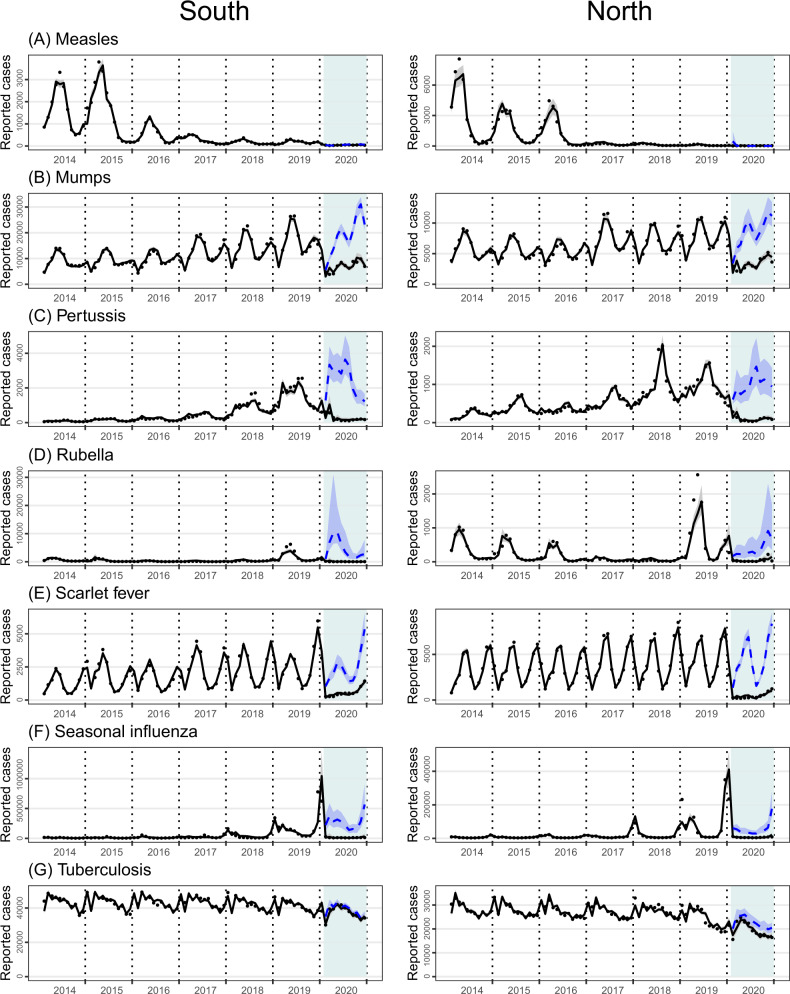
Time series of observed (black dots) and GLM-projected monthly numbers of reported cases for selected respiratory diseases. **A** Measles, **B** mumps, **C** pertussis, **D** rubella, **E** scarlet fever, **F** seasonal influenza, **G** tuberculosis. The model-projected trajectories are shown for both with (black solid) and without (blue dash, 2020 only) nonpharmaceutical interventions. Intervention phases II–IV (Feb–Dec) in 2020 are colored light blue. For the counterfactual trajectory without NPIs, monthly volumes of outpatient visits in 2020 were assumed the same as in 2019.

**Fig. 3:**
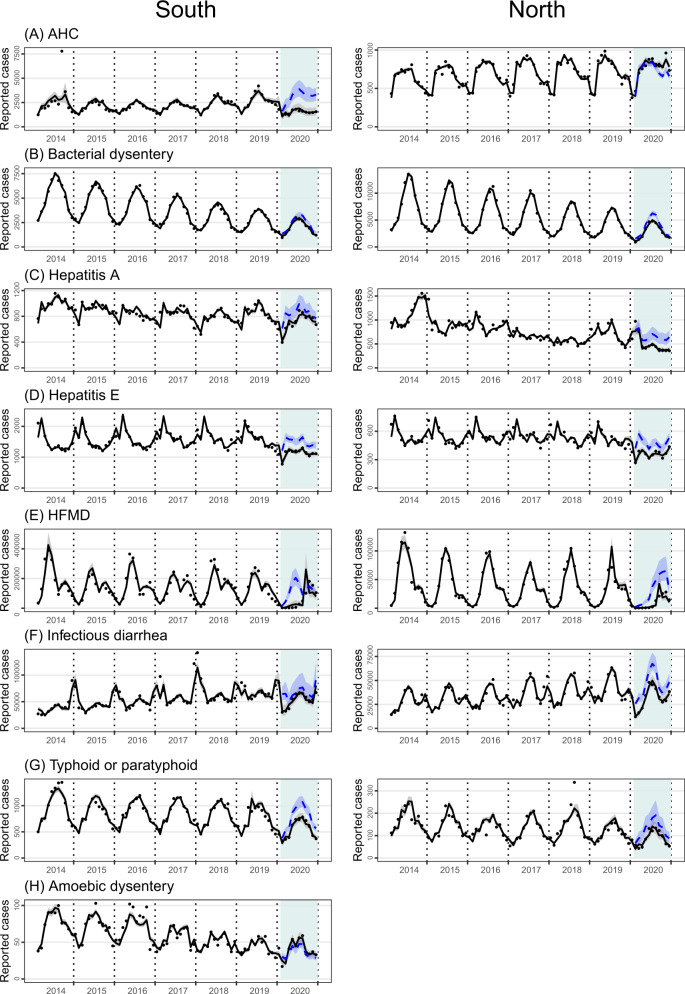
Time series of observed (black dots) and GLM-projected monthly numbers of reported cases for selected gastrointestinal or enteroviral diseases. **A** Acute hemorrhagic conjunctivitis, **B** bacterial dysentery, **C** hepatitis A, **D** hepatitis E, **E** hand, foot, and mouth disease, **F** infectious diarrhea, **G** typhoid or paratyphoid, and **H** amebic dysentery. The model-projected trajectories are shown for both with (black solid) and without (blue dash, 2020 only) nonpharmaceutical interventions. Intervention phases II–IV (Feb–Dec) in 2020 are colored light blue. For the counterfactual trajectory without NPIs, monthly volumes of outpatient visits in 2020 were assumed the same as in 2019.

Most notifiable diseases were significantly reduced during at least one of the pandemic phases II–IV in 2020 compared with 2014–2019, with estimated incidence-rate ratios (IRRs) below1 and *p*-values <0.05, except for measles, amebic dysentery, brucellosis, and typhus (Table 2). NPIs appeared more influential on respiratory diseases such as mumps, pertussis, rubella, scarlet fever, and seasonal influenza, with IRR estimates ranging from 0.01 (95% confidence intervals: 0.004−0.06) to 0.53 (0.39−0.71) in the south and from 0.05 (0.03−0.08) to 0.48 (0.35−0.65) in the north (Table 2). The monthly incidences of HFMD were tremendously reduced during phases II and III in both the south and the north, with IRR estimates ranging from 0.10 (0.02−0.49) to 0.20 (0.10−0.42). The incidence of malaria was reduced during all three pandemic phases, with IRR estimates ranging from 0.12 (0.08−0.17) to 0.62 (0.45−0.85). Many other respiratory diseases, gastrointestinal or enteroviral diseases, sexually transmitted or blood borne diseases, and vector-borne or zoonotic diseases also showed statistically significant reductions, though to a less extent. Reductions were generally more prominent in Phase II than in Phase III for nonrespiratory diseases, but for respiratory diseases, the opposite was observed, as more drastic reductions were associated with Phase III and carried forward to Phase IV (Table 2). Resurgence to beyond historical levels was seen for AHC in the north, and brucellosis in the whole nation. Monthly incidences of hepatitis A, HFMD, infectious diarrhea, hepatitis B, and hepatitis C also rose in phases III or IV, close to historical levels. Measles, amebic dysentery, HIV/AIDS, typhus, and hydatid disease seemed to be less impacted by NPIs. The potential impact of NPIs is more clearly seen in the differences between the observed monthly numbers of reported cases and the model-projected (counterfactual) counts had there been no NPI in place (Figs. 2–3, Supplementary Fig. 16–17). For example, all respiratory diseases, except for measles and TB, showed a large number of potential cases likely averted by the NPIs during 2020. The gap between model-projected and observed epidemic curves reflected changes of both the healthcare-seeking behavior and disease incidence, as we used the volumes of outpatient visits in 2019 for projection. We performed a sensitivity analysis using harmonic functions instead of SEATS to adjust for seasonality, and the results are qualitatively similar to the primary analysis (Supplementary Figs. 18–21, Supplementary Table 2).

## Discussion

Using the national surveillance data of notifiable infectious diseases in China during the last seven years, the current study characterized the change patterns of a variety of infectious diseases in association with the implementation and subsequent relaxation of NPIs intended to mitigate the COVID-19 pandemic. By comparing incidences in 2020 to the average historical levels during 2014–2019 within each phase defined by the timeline of NPIs, we found that the spread of most acute infectious diseases, especially respiratory and gastrointestinal/enteric diseases, was largely interrupted during February–August (phases II–III) of 2020 when the most stringent NPIs were in effect. Respiratory diseases were more sensitive to the NPIs than other disease categories. Several gastrointestinal/enteric and zoonotic diseases, such as HFMD and infectious diarrhea disease, rebounded quickly when NPIs were relaxed.

COVID-19-related NPIs likely affected the transmission of other infectious diseases mainly through reducing human-to-human contact (affecting respiratory diseases, gastrointestinal or enteroviral diseases, and sexually transmitted or bloodborne diseases) and environmental exposure (affecting gastrointestinal or enteroviral diseases and vector-borne or zoonotic diseases). Soon after Wuhan’s lockdown was lifted in April, NPIs were gradually relaxed and socioeconomic activities resumed nationwide, leading to resurgence of some diseases in Phase IV. The most typical example is HFMD. As a childhood disease, HFMD usually peaks in spring or early summer, which corresponds to Phases II and III^17^. As schools and daycares were closed during this time in 2020, HFMD incidences plunged. The rebound of HFMD in Phase IV was likely a result of reopening of schools and daycares in September. In contrast, the incidence of the respiratory diseases remained low till the end of the year, which might be attributable to the persistent nationwide policy of wearing face masks in public spaces including schools^18^. Measles is the only respiratory disease that did not show significant change in the GLM, likely due to its very low incidences under a high-vaccination coverage in all provinces in recent years (Fig. 2)^19^. However, the change pattern of respiratory diseases in response to the COVID-19 NPIs could vary from country to country, depending on the nature and scope of the NPIs. For example, one study conducted in the United Kingdom showed that the circulation of rhinoviruses was remarkably increased once schools reopened^20^. Inadequate social distancing and lack of mandated mask-wearing likely contributed to the increased school transmission of rhinovirus in the United Kingdom. Unfortunately, rhinovirus is not a notifiable infectious disease in China, thus, we cannot assess its change pattern in the current study.

Less variation of sexually transmitted or bloodborne diseases was observed in comparison with respiratory diseases and gastrointestinal or enteroviral diseases, possibly due to the difference in their transmission modes. However, the incidences of most sexually transmitted or blood-borne diseases did decline, particularly in Phase II when human movement was strictly restricted. Unlike other sexually transmitted or bloodborne diseases, gonorrhea, an acute disease, showed a sharp increase to surpass historical levels in phases III and IV in southern China. In recent years, gonorrhea features high incidence in southern provinces of China, particularly among young adults in the following sectors: farmers, unemployed, business, and industrial workers^21^. The rebound might be related to the lifting of travel restrictions and renewal of socioeconomic activities.

NPIs likely modulated the incidences of vector-borne or zoonotic diseases in at least two more ways in addition to altering healthcare-seeking behavior. The restrictions on human mobility inevitably reduced people’s outdoor activities and hence lowered their exposure to vectors and animal hosts of vector-borne or zoonotic diseases. Meanwhile, restrictions and screening of international travelers dramatically reduced the number of imported cases. Dengue fever and malaria, two mosquito-borne diseases, are examples of the latter mechanism. The two diseases are currently not endemic in China but mainly imported by international travelers in winter seasons. The incidences of the two diseases rose in Phase I of 2020, when the Chinese Lunar New Year was approaching and international travel volume was peaking, and plunged in Phase II with the tightened border control. Low incidences persisted though the end of the year as border control remained tight. For locally endemic vector-borne or zoonotic diseases such as Japanese encephalitis, changes could be explained by fewer outdoor activities of human hosts and frequent indoor and outdoor disinfections that might have reduced mosquito density^22,23^. The resurgence of zoonotic diseases such as brucellosis and typhus could be a result of the recovering work activity of herdsmen and farmers. Educational programs and personal-protection equipment are needed to reduce their exposure.

The impact of NPIs on disease incidences also varied geographically, probably a consequence of heterogeneity in culture, population density, contact behavior, and surveillance capacity. In provinces where local COVID-19 outbreaks recurred later in 2020, i.e., Heilongjiang, Xinjiang and Liaoning, NPIs were reinforced and the reported incidences of many notifiable diseases remained below historical levels throughout the rest of the year^24–26^.

We noticed significant changes in health-seeking behavior during the pandemic, which had partially contributed to the reduction in the reported-disease incidences. On the other hand, we observed a stable trend in the proportion of laboratory confirmation among all reported cases throughout recent years, including 2020, indicating sustained and effective surveillance of notifiable diseases, despite possible diversion of medical resources to COVID-19 testing in 2020.

Our study has some limitations. First, this is an observational study; thus, our results should be interpreted as associations of COVID-19 NPIs with, rather than causal effect on, reducing transmission of non-COVID-19 infectious diseases. Decreased disease incidences could be partly driven by altered healthcare-seeking behaviors and surveillance capacity by the pandemic, an inevitable consequence of human-mobility restrictions and overstressed healthcare facilities. Such bias could be more severe in the early wave (e.g., Phase II) of the pandemic. Nevertheless, we have used the monthly outpatient volume as a surrogate to partially offset the impact of altered healthcare-seeking behaviors. The proportions of lab-confirmed cases among all reported cases remained relatively stable over time and space for most notifiable diseases, suggesting that surveillance of notifiable diseases remained effective, despite possible diversion of medical resources to COVID-19 testing in 2020. In the future, surveillance bias of infectious diseases may be better assessed if more health seeking or delivery indicators become available, e.g., incidences of noncommunicable diseases, routine health screening, and use of over-the-counter pharmaceuticals. Second, the spatial heterogeneity in disease patterns and the effect of NPI measures might have been correlated with socioenvironmental factors such as population density, age structure, weather conditions, transportation, and compliance with NPIs, which we did not evaluate in the current study. Third, assessment of the effect of any single NPI was not pursued because the NPIs were implemented collectively and their timelines were similar across most provinces. In addition, improved diagnostic technology for some infectious disease in recent years could be another source of bias in the results. For example, the wide application of rapid influenza-test reagent in hospitals since 2018 had led to a sharp increase in diagnosed influenza cases afterward, which could have affected the comparison of Phase I between 2020 and previous years. Last, the monthly data were used for modeling which do not fully align with the exact timeline of NPIs, but the differences are relatively small.

Our study highlights the broad-spectrum correlations of NPIs with the spread of infectious diseases. However, strongly disruptive NPIs such as travel and gathering bans are generally not affordable as a long-term solution for their negative impact on economic and social activities. A combination of less disruptive NPIs, such as social distancing and mask-wearing for large indoor gathering and mass-transportation vehicles, with efficacious vaccines and therapies, is likely a better solution than relying on either one alone. For the diseases insensitive to less-disruptive NPIs such as HFMD, development and evaluation of new NPIs should be encouraged. Finally, the variation of reported incidences of chronic infectious diseases such as HIV/AIDS and TB could be more related to underreporting than to the NPIs, and potential future increase in disease progression and mortality due to delayed diagnosis and treatment during the pandemic needs to be closely monitored^27,28^.

## Methods

### The surveillance system

China Information System for Disease Control and Prevention (CISDCP) is an Internet-based real-time disease-reporting system covering 40 notifiable diseases (COVID-19 was included since January 2020)^29^. First established in 2004, this system has evolved to cover 55 077 national health facilities in 397 cities of all 31 provinces in the mainland of China. Upon clinical or laboratory diagnosis in accordance with guidelines issued by the National Health Commission of the People’s Republic of China (http://www.nhc.gov.cn), standardized data of individual cases are electronically transmitted from hospitals, local Centers for Disease Control and Prevention (CDCs), community health centers, township health centers, and village clinics to the central database located at the China CDC within 24 h^30^. All notifiable infectious diseases were diagnosed according to their standard diagnostic criteria^30^. Only patients with confirmed diagnosis (lab-confirmed and clinically confirmed cases) were included in the analysis and the suspected cases were excluded. Patients of COVID-19 are also reported to the CISDCP, and the diagnosis is based on the COVID-19 Diagnosis and Treatment Protocol issued by the National Health Commission. All patients with fever or respiratory symptoms are required to visit designated hospitals that are capable of diagnosing COVID-19 and other notifiable infectious diseases. According to the protocol of reporting notifiable infectious diseases^31^, each diagnosed case is assigned a unique resident ID number in the CISDCP. Demographic data such as name, sex, age, and occupation, clinical data such as dates of symptom onset, diagnosis, hospital visit, and death, and laboratory results (if available) are first logged into hospital electronic health record systems by physicians or trained hospital staff, which are reviewed and formally uploaded to the CISDCP by the staff. Data uploaded by hospitals will be further verified by staff at local CDC for mistakes, omissions, duplications, and laboratory test (if patient’s samples were collected and laboratory-tested by local CDC). If the laboratory result or clinical status (such as death) of a patient becomes available at a later stage, the updated data will be submitted by hospital staff and verified by local CDC, and the mechanism is in place to avoid duplication of records during the updating process. In addition to patient data, reporting physician and hospital, and dates of reporting, updating, review, and finalization are also recorded by the CISDCP.

### Data collection

Daily case numbers of 31 notifiable infectious diseases at the province level during 2014–2020 were extracted from the CISDCP. Diseases with less than 2000 reported cases during this period (filaria, hepatitis D, diphtheria, neonatal tetanus, meningococcal meningitis, cholera, plague, kala-azar, poliomyelitis, avian influenza H7N9, H5N1, H1N1, and SARS), and leprosy (direct contact transmission) were excluded. The remaining 31 diseases were further grouped into four categories: respiratory diseases, gastrointestinal or enteroviral diseases, sexually transmitted or bloodborne diseases, and vector-borne or zoonotic diseases (Supplementary Table 3). For each reported case, we extracted age, sex, occupation, city of residence, date of symptom onset, severity of symptoms at diagnosis, type of diagnosis (clinical or laboratory), date of diagnosis, clinical outcome (recovery or death), and date of death if the outcome is death. Demographic statistics at the province level were collected from the National Bureau of Statistics of China (www.stats.gov.cn). Monthly outpatient-visit volumes were collected from the Center for Health Statistics and Information (http://www.nhc.gov.cn/mohwsbwstjxxzx/s2906/new_list.shtml). It was determined by the National Health Commission of China that the collection of data on notifiable infectious diseases was part of continuing public health surveillance of infectious diseases and was exempt from Institutional Review Board assessment. All the data of cases used in this study were anonymized and personal identification information (e.g., name and street address) is not included in the data, and this study was approved by the Chinese Center for Disease Control and Prevention (202026).

### Statistical analysis

To investigate the impact of nonpharmaceutical interventions, we define four epidemic phases (Supplementary Fig. 22), Jan 1–Jan 22 (Phase I), Jan 23–Apr 7 (Phase II), Apr 8–Aug 31 (Phase III), and Sep 1–Dec 31 (Phase IV), according to the timeline of major intervention events for containing the COVID-19 epidemic during 2020 in China. Phase I marks the period without NPIs. Phase II started with the lockdown of Wuhan on Jan 23, during which all provinces initiated level-1 response to the public health emergency which means enforcing most intense NPIs such as stay-at-home or shelter-in-place order^32^, closure of nonessential businesses, restaurant, schools and hotels, prohibition of gatherings, etc. In Phase III, all provinces downgraded their public health response to level 2 or level 3. These lower levels reopened public transportation, some higher educational institutes, and certain businesses, but stringent NPIs still remained, e.g., social distancing, routine temperature monitoring, and limiting the capacity of businesses. Sep 1 marks the start of Phase IV, when schools reopened and businesses and recreational activities resumed nationwide.

Bar plots were used to compare disease incidences of 2020 to the average incidences of 2014–2019 in each of the four phases. For each disease, percent change (PC) of incidence was calculated for each province as well as for the country as

1 $$\left[({{{{{\rm{inc}}}}}}_{2020}(k))-{{{{{\rm{inc}}}}}}_{2014-2019}(k)\right]/{{{{{\rm{inc}}}}}}_{2014-2019}(k)\times 100 \%$$

where ${{{{{\rm{inc}}}}}}_{2014-2019}(k)$ indicates the average incidence during phase *k* over 2014–2019 and ${{{{{\rm{inc}}}}}}_{2020}(k)$ indicates the incidence specific to phase *k* of 2020. Phase-specific incidences (/10^5^ people) and PCs were plotted by disease, year, or demographic group when needed. Error bars were also plotted for PCs but not for incidence rates as they are too narrow to be visible.

Relative change of monthly outpatient-visit volume in 2020 compared with 2019 was calculated as:

2 $$({{{{{\rm{vol}}}}}}_{2019,i}-{{{{{\rm{vol}}}}}}_{2020,i})/{{{{{\rm{vol}}}}}}_{2019,i}\times 100 \%$$

where the ${{{{{\rm{vol}}}}}}_{t,i}$ indicates the volume of outpatient visits in month $i$ of year $t$. The impact of healthcare-visit volume change was assessed quantitatively by comparing to the relative changes in the number of reported cases during the same month $i$. The 95% CIs of percent change were calculated by delta method^33^.

To assess the stability of the surveillance-system operation during the COVID-19 pandemic, the proportion of laboratory-confirmed cases among all confirmed cases for each infectious disease was calculated by year and by provincial level.

To adjust for potential confounders of NPI effect, e.g., changes in the volume of outpatient visits and long-term disease trend, a series of multivariable generalized linear models (GLM) were applied to quantify the impact of NPIs on each notifiable disease in southern and northern China separately. Diseases with a median monthly number of reported cases <50 during 2014–2020 were excluded. As monthly case numbers were being modeled, we redefined the pandemic phases as Phase I (Jan), Phase II (Feb and Mar), Phase III (Apr–Aug), and Phase IV (Sep–Dec), which is largely consistent with the previous definition. We first used the X-13ARIMA-SEATS (Signal Extraction in ARIMA Time Series) method^34^ to obtain seasonality-removed monthly case numbers for each disease. SEATS estimates seasonality while accounting for potential shifts in the mean level of the time series, e.g., the downward shift in disease incidence due to the implementation of NPIs during 2020. We then fitted GLMs to the seasonality-removed monthly case numbers in two stages. In stage 1, a quasi-Poisson model was fitted with the following predictors: phase indicators for the year 2020, long-term trend, number of person-days (population size times the number of days in the month) as offset, and monthly volume of outpatient visits. In stage-2, the same model as in stage 1 was used, with residuals from stage 1 model at one-month lag as an additional predictor to account for autocorrelation. Incidence rate ratios (IRR) associated with the phase indicators estimated by the stage 2 model then reflect the effects of COVID-19 NPIs on the incidence of each notifiable disease in 2020^35,36^. The quasi-Akaike information criterion (QAIC) was examined for model selection, e.g., the choice of long-term trend (flat, linear, or quadratic, Supplementary Table 4). A two-sided *P* < 0.05 was considered statistically significant. Multiplying the seasonality estimated by the X-13ARIMA-SEATS back to the fitted GLMs, we then projected counterfactual monthly case numbers in the absence of NPI. As NPIs had also affected the healthcare-seeking behavior, for projection, we assumed that the monthly outpatient-visit volumes in 2020 were the same as those in 2019. More details about the GLM analysis can be found in the supplementary information. In addition, we did a sensitivity analysis by using the harmonic functions instead of the X-13ARIMA-SEATS to adjust for seasonality (detailed in the supplementary information). All statistical analyses were conducted in R software (version 4.0.3, R Development Core Team 2020).

### Reporting summary

Further information on research design is available in the Nature Research Reporting Summary linked to this article.

## Supplementary information

Supplementary Information

Description of Additional Supplementary Files

Supplementary Data 1

Reporting Summary

## Data Availability

The relevant data of the model are provided in the supplementary information. Raw data are not publicly available and are protected due to data privacy laws, which were used under license for the current study, but are available upon reasonable request to the corresponding author and with permission from the data provider (Li-Ping Wang). The request will be responded within one week.

## Code availability

The R code of the models is provided in Supplementary Data 1.

## References

[1] Markel H (2007). Nonpharmaceutical interventions implemented by US cities during the 1918–1919 influenza pandemic. JAMA.

[2] Cauchemez S, Valleron AJ, Boëlle PY, Flahault A, Ferguson NM (2008). Estimating the impact of school closure on influenza transmission from Sentinel data. Nature.

[3] Mitchell T (2011). Non-pharmaceutical interventions during an outbreak of 2009 pandemic influenza A (H1N1) virus infection at a large public university, April-May 2009. Clin. Infect. Dis..

[4] Loustalot F (2011). Household transmission of 2009 pandemic influenza A (H1N1) and nonpharmaceutical interventions among households of high school students in San Antonio, Texas. Clin. Infect. Dis..

[5] Teasdale E (2014). Public perceptions of non-pharmaceutical interventions for reducing transmission of respiratory infection: systematic review and synthesis of qualitative studies. BMC Public Health.

[6] Blackley DJ (2015). Rapid intervention to reduce Ebola transmission in a remote village—Gbarpolu County, Liberia, 2014. MMWR Morb. Mortal. Wkly. Rep..

[7] Olsen SJ (2020). Decreased influenza activity during the COVID-19 pandemic - United States, Australia, Chile, and South Africa, 2020. MMWR Morb. Mortal. Wkly. Rep..

[8] Yeoh DK (2020). The impact of COVID-19 public health measures on detections of influenza and respiratory syncytial virus in children during the 2020 Australian winter. Clin. Infect. Dis..

[9] Baker RE (2020). The impact of COVID-19 nonpharmaceutical interventions on the future dynamics of endemic infections. Proc. Natl Acad. Sci. USA.

[10] Auger KA (2020). Association between statewide school closure and COVID-19 incidence and mortality in the US. JAMA.

[11] Huang QS (2021). Impact of the COVID-19 nonpharmaceutical interventions on influenza and other respiratory viral infections in New Zealand. Nat. Commun..

[12] Huang F (2020). The impact of the COVID-19 epidemic on tuberculosis control in China. Lancet Reg. Health West. Pac..

[13] Kraay ANM (2021). Impact of nonpharmaceutical interventions for severe acute respiratory syndrome coronavirus 2 on norovirus outbreaks: An analysis of outbreaks reported by 9 US states. J. Infect. Dis..

[14] Lei H (2020). Nonpharmaceutical interventions used to control COVID-19 reduced seasonal influenza transmission in China. J. Infect. Dis..

[15] Wong NS, Leung CC, Lee SS (2020). Abrupt subsidence of seasonal influenza after COVID-19 outbreak, Hong Kong, China. Emerg. Infect. Dis..

[16] Yang S (2017). Epidemiological features of and changes in incidence of infectious diseases in China in the first decade after the SARS outbreak: an observational trend study. Lancet Infect. Dis..

[17] Hu M (2012). Determinants of the incidence of hand, foot and mouth disease in China using geographically weighted regression models. PLoS ONE.

[18] Ministry of Education of the People’s Republic of China. *COVID-19 prevention and control guidelines for kindergartens, primary and secondary schools and universities* (2020); http://www.moe.gov.cn/jyb_xwfb/gzdt_gzdt/s5987/202003/t20200312_430163.html

[19] Zuo S (2020). Increasing vaccination coverage: the school entry vaccination record check program in Guizhou Province China, 2003–2018. Vaccine.

[20] Poole S, Brendish NJ, Tanner AR, Clark TW (2020). Physical distancing in schools for SARS-CoV-2 and the resurgence of rhinovirus. Lancet Resp. Med..

[21] Yue XL, Gong XD, Li J, Wang YJ, Gu H (2019). Gonorrhea in China, 2018. Int. J. Dermatol. Venereol..

[22] Health Commission of Guangdong Province. *The density of Aedes mosquitoes, vectors of dengue and Zika viruses, in the first half of July 2020* (2020); http://wsjkw.gd.gov.cn/gkmlpt/content/3/3054/post_3054335.html#257

[23] Health Commission of Guangdong Province. *The density of Aedes mosquitoes, vectors of dengue and Zika viruses, in the second half of July 2020* (2020); http://wsjkw.gd.gov.cn/gkmlpt/content/2/2585/post_2585753.html#2571

[24] Health Commission of Xinjiang. *COVID-19 pandemic information in Xinjiang in August* (2020); http://wjw.xinjiang.gov.cn/hfpc/tzgg/zfxxgk_gknrz.shtml

[25] Health Commission of Liaoning. *COVID-19 pandemic information in Liaoning in August* (2020); http://wsjk.ln.gov.cn/wst_wsjskx/

[26] Health Commission of Heilongjiang. *COVID-19 pandemic information in Heilongjiang in August* (2020); http://wsjkw.hlj.gov.cn/pages/5df84bfaf6e9fa23e8848a48

[27] World Health Organization. *Global tuberculosis report 2020* (2020); https://apps.who.int/iris/handle/10665/336069

[28] Hogan AB (2020). Potential impact of the COVID-19 pandemic on HIV, tuberculosis, and malaria in low-income and middle-income countries: a modelling study. Lancet Glob. Health.

[29] Wang L (2008). Emergence and control of infectious diseases in China. Lancet.

[30] Dong Y (2020). Infectious diseases in children and adolescents in China: analysis of national surveillance data from 2008 to 2017. BMJ.

[31] Chinese Center for Disease Control and Prevention. *Protocol of infectious disease reporting* (2020); http://www.chinacdc.cn/jkzt/crb/xcrxjb/201810/t20181017_195160.html

[32] Fang LQ (2020). Meteorological conditions and nonpharmaceutical interventions jointly determined local transmissibility of COVID-19 in 41 Chinese cities: a retrospective observational study. Lancet Reg. Health West. Pac..

[33] Miguel Angel Luque Fernandez. *Delta Method in Epidemiology: An Applied and Reproducible Tutorial* (2020); https://migariane.github.io/DeltaMethodEpiTutorial.nb.html

[34] Sax C, Eddelbuettel D (2018). Seasonal adjustment by X-13ARIMA-SEATS in R. J. Stat. Softw..

[35] Brumback BA (2000). Transitional regression models, with application to environmental time series. J. Am. Stat. Assoc..

[36] Imai C, Armstrong B, Chalabi Z, Mangtani P, Hashizume M (2015). Time series regression model for infectious disease and weather. Environ. Res..

