# Supplementary material for: Changes in notifiable infectious disease incidence in China during the COVID-19 pandemic
Source: Nat Commun. 2021 Nov 26;12:6923. doi: 10.1038/s41467-021-27292-7 (PMC8626444; doi:10.1038/s41467-021-27292-7)
Supplement: Supplementary file 1 — Supplementary Information [file 41467_2021_27292_MOESM1_ESM.pdf]

Supplementary information

Supplement to: Changes in Notifiable Infectious Diseases Incidence in China during the COVID-19 Pandemic

Supplementary Methods

Data collection and classification of the notifiable infectious diseases

In the China Information System for Disease Control and Prevention (CISDCP), viral hepatitis is further grouped into hepatitis A, hepatitis B, hepatitis C, hepatitis D, hepatitis E and untyped hepatitis, transmission routes and clinical manifestations of which vary. Similarly, dysentery is divided into bacterial dysentery and amoebic dysentery, respectively. In total, 46 kinds of infectious diseases (COVID-19 was added in 2020) are reported in CISDCP,[1] among which fifteen (filaria, hepatitis D, untyped hepatitis, diphtheria, neonatal tetanus, meningococcal meningitis, cholera, plague, kala-azar, leprosy, poliomyelitis, H7N9, H5N1 and SARS) were excluded from the current analysis due to low incidence rates. COVID-19 was also excluded due to the purpose of this study. The remaining 31 infectious diseases were included in our analysis. Infectious diarrhea diseases other than cholera, bacterial dysentery, amoebic dysentery, typhoid and paratyphoid were combined to be analyzed as a single disease.

NPIs implemented during the pandemic year 2020

We define four intervention phases in line with the level of the public health emergency response that varied over time, with a higher level corresponding to more restrictions. Three-level responses were implemented during the COVID-19 pandemic in China. Level 1 response is the highest level, including human movement restrictions (stay-at-home or shelter-in-place), closure of nonessential businesses, closure of restaurants and hotels, closure of all schools (daycares, elementary, middle and high schools, professional schools, and colleges), and prohibition of public gatherings. Level 2 response relaxes human movement restrictions in areas without cases and allows restaurants to operate with limited capacity. Schools remain closed and indoor gathering activities (such as cinema) are prohibited. Level 3 response differs from Level 2 in that schools reopen, business activities resume, and public gathering is allowed with prevention and self-protection such as monitoring temperature and wearing face masks. In Phase I, no formal NPI was initiated. During Phase II, Level 1 response was put in place in all provinces. During Phase III, most provinces transitioned to Level 2 and then to Level 3 gradually for economic activities to recover. During Phase IV, Level 3 response continued in all provinces. The exact measures in each level of response varied slightly across provinces but were largely similar. By April 8, the first wave essentially ended with no province reporting new cases, although sporadic outbreaks recured later of the year in Heilongjiang, Xinjiang and Liaoning.

Generalized Linear Models

In the modeling analysis, we assess the association of NPIs with disease incidences while accounting for changes in healthcare seeking behavior and autocorrelation among the case numbers. We use the monthly volume of outpatient visits as a surrogate for healthcare seeking behavior. Outpatient data of Hubei and Tibet are not available and therefore the two provinces are excluded from the GLM analysis. As outpatient data are available only at the monthly scale, the outcome of the GLM is the monthly case numbers, and we redefine the intervention phases as Phase I (Jan), Phase II (Feb and Mar), Phase III (Apr−Aug) and Phase IV (Sep−Dec), which is largely consistent with the previous definition. The modeling steps are conducted as follows for each specific notifiable infectious disease in South China and North China.

(1). We first use X13ARIMA-SEATS, a time series decomposition approach, to decompose the time series of monthly reported case numbers into a seasonal component and a nonseasonal component, and we refer to the latter as seasonality-removed numbers of reported cases.[2] Briefly, SEATS (signal extraction in ARIMA time series) is a seasonal adjustment tool based on ARIMA model decomposition of time series data.[3] SEATS decomposes the linearized time series into trend, seasonal, transitory and irregular components, provides forecasts for these components together with associated standard errors, and assign deterministic effects to each component. A fundamental assumption made by SEATS is that the linearized time series y_t (which is the log of monthly case number in our analysis) follows the ARIMA model

$$\phi(B)\Phi(B_s)(1-B)^d(1-B_s)^D(y_t-\boldsymbol{x}_t{}'\boldsymbol{\beta})=\theta(B)\Theta(B_s)a_t$$

where y_t is the time series, $\boldsymbol{x}_t{}'\boldsymbol{\beta}$ is the regression part with covariates $\boldsymbol{x}_t$, a_t is the white noise with mean 0 and variance σ^2, and

B and B_s: the non-seasonal and seasonal backshift operators, i.e., $B(y_t)=y_{t-1}$, $B_s(y_t)=y_{t-12}$;

$\phi(B)=1-\phi_1 B-\cdots-\phi_p B^p$: a non-seasonal autoregressive (AR) operator of order p;

$\Phi(B_s) = 1 - \phi_1 B_s - \cdots - \phi_P {B_s}^P$: a seasonal autoregressive operator of order P;
$(1-B)^d$ and $(1-B_s)^D$: non-seasonal and seasonal differencing operators of orders d and D, respectively;
$\theta(B) = 1 - \theta_1 B - \cdots - \theta_q B^q$: a non-seasonal moving average (MA) of order q;
$\Theta(B_s) = 1 - \Theta_1 B - \cdots - \Theta_Q {B_s}^Q$: a seasonal moving average of order Q;

We perform the decomposition using the SEATS implemented in the R package "seasonal" (version 1.8.3).[4] For the regression part, we only consider the holiday effect of Chinese New Year generated by the "genhol" function. One nice feature of SEATS is that it automatically detects shifts in the mean level of the time series, suggesting that it can partially account for the impact of NPIs during 2020 when estimating seasonality. We use the default setting for the selection of the orders of all operators using the Bayesian Information Criterion (BIC) provided by "seasonal". Inference of model parameters is based on a combination of the iterative least square (IRLS) step for the regression parameters $\boldsymbol{\beta}$ and maximum likelihood for other parameters, where the maximum likelihood estimation is based on the standard approach by Box and Jenkins (1976).[5] Two outputs are obtained from SEATS, the seasonality-removed monthly case numbers and the seasonal trend itself. The seasonal trend is then extrapolated to 2020 to obtain seasonality-removed case numbers during the pandemic.

(2). A generalized linear model is fitted to the seasonality-removed monthly case numbers, which we assume follow a quasi-Poisson distribution to account for overdispersion. The following covariates are adjusted in the model:

(i) Three binary indicators for COVID-19 NPI phases during 2020: phase II for Feb−Mar 2020, Phase III for Apr−Aug 2020, and phase IV for Sep−Dec 2020. Other months from Jan 2014 to Jan 2020 serve as the reference when no NPIs was implemented.

(ii) Monthly volume of outpatient visits. Outpatient visit data in December of every year were not available and we imputed these numbers with the average between the adjacent November and January.

(iii) Long-term effect as a function of year, which can be none, linear or quadratic selected by the quasi-Akaike information criterion (QAIC). The functional form with the smallest QAIC was selected (Supplementary Table 4).

(iv) The product of the number of days in each month and the population size was used as the offset.

Residuals of the fitted model were calculated. The GLM and residuals obtained are referred to as the stage-1 GLM and stage-1 residuals, respectively.

(3). Another GLM is fitted to the seasonality-removed case numbers, which is the same as the GLM above except that stage-1 residuals were included as a covariate, with a one-month lag, to account for autocorrelation in the outcome.[6] Residuals from this model are calculated and auto-correlation function is plotted to assess whether autocorrelation has been adequately removed (see supplementary Figs. 23−26). The GLM and residuals obtained are referred to as the stage-2 GLM and stage-2 residuals, respectively. A 95% confidence band for model-fitted cases counts is constructed by bootstrapping model coefficients from the asymptotic distribution (multivariate normal) of the coefficient estimates.

(4). Finally, the seasonal component estimated by X13ARIMA-SEATS is multiplied (the seasonal trend is estimated at the log scale in step 1) back to the fitted stage-2 GLM to project counterfactual numbers of reported cases during 2020 had the COVID-19 NPIs been absent. As healthcare seeking behavior was also altered by the pandemic, we assume the outpatient visit data in 2020 were the same as in 2019 for the projection of 2020. The stage-1 residuals are also dropped from the covariates of the stage-2 GLM for projection. To construct s 95% confidence band for the projected trajectory, we sample both coefficient from their asymptotic distribution and residuals from the stage-1 residuals associated with months during 2014 to 2019. The residuals are sampled in blocks of 10 months to preserve the autocorrelation structure. Sampled residuals are then added to the model-projected values to form the confidence band.

Sensitivity Analysis

As an alternative adjustment for seasonal trends, we skip the SEATS decomposition step but incorporate harmonic functions into stage-1 GLM and fit the model to the original data to obtain seasonality-adjusted stage-1 residuals. We consider harmonic functions with annual or semiannual seasonal cycles, and the exact number of harmonics is chosen by QAIC for each pathogen and region. Then, a stage-2 GLM is fitted to the original case numbers, which is the same as the stage-1 GLM except that stage-1 residuals are included as a covariate, with a one-month lag, to account for autocorrelation. We show the sensitivity results in Supplementary Figs. 18−21 and Supplementary Table 2, which are qualitatively similar to

the primary analysis. Interestingly, SEATS allows slight variation of seasonality from year to year, in comparison to invariant seasonality enforced by harmonic functions, e.g., as seen by comparing the model-projected mumps epidemic curves during 2020 between Figure 2 (SEATS+GLM) and Supplementary Figure 18 (GLM with harmonic functions).

We also considered a single GLM including not only long-term trend and harmonic functions but also an autoregressive term of the case number from the previous month, $\log(Y_{t-1})$.[7] However, this model is unlikely to yield reliable results (not shown) mostly due to the high correlation between the autoregressive term and the temporal trend combining the long-term trend and harmonic functions (Supplementary Table 5).

In addition, we performed a sensitivity analysis using a single-stage GLM that only compares incidence in the four periods between 2014−2019 and 2020, without adjusting for monthly healthcare visits, yearly long-term trend, seasonality or autocorrelation (Supplementary Table 6). This unadjusted model detected fewer differences in respiratory diseases and Gastrointestinal or enteroviral disease than the adjusted model in Table 2 of the main text.

Supplementary Figure 1. The spatial grouping of 31 mainland provinces into North China (blue) and South China (red).

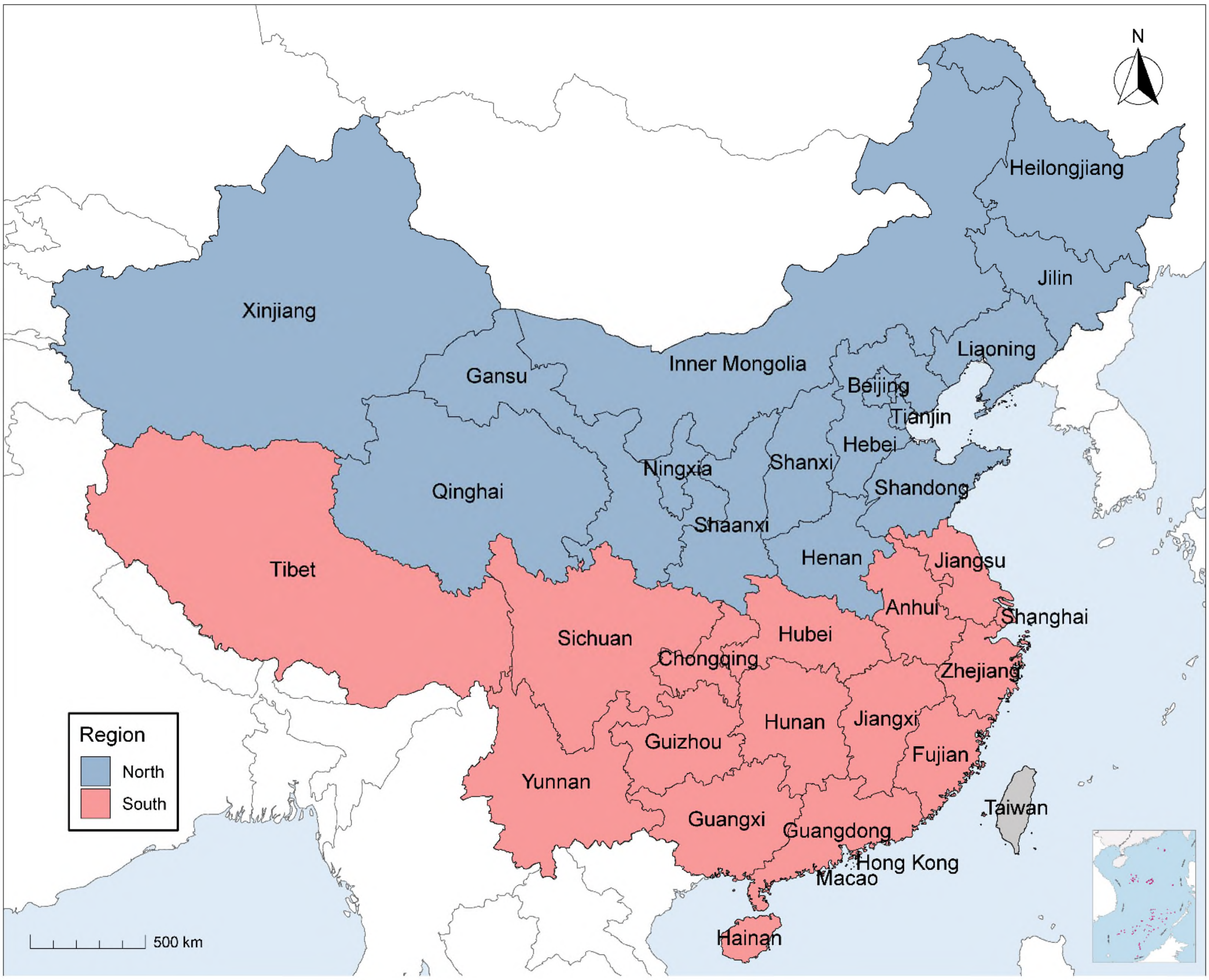

Supplementary Figure 2. The annual proportions of lab confirmed (dark) and clinically confirmed (light) cases among all reported cases of notifiable diseases in China from 2014 to 2020.

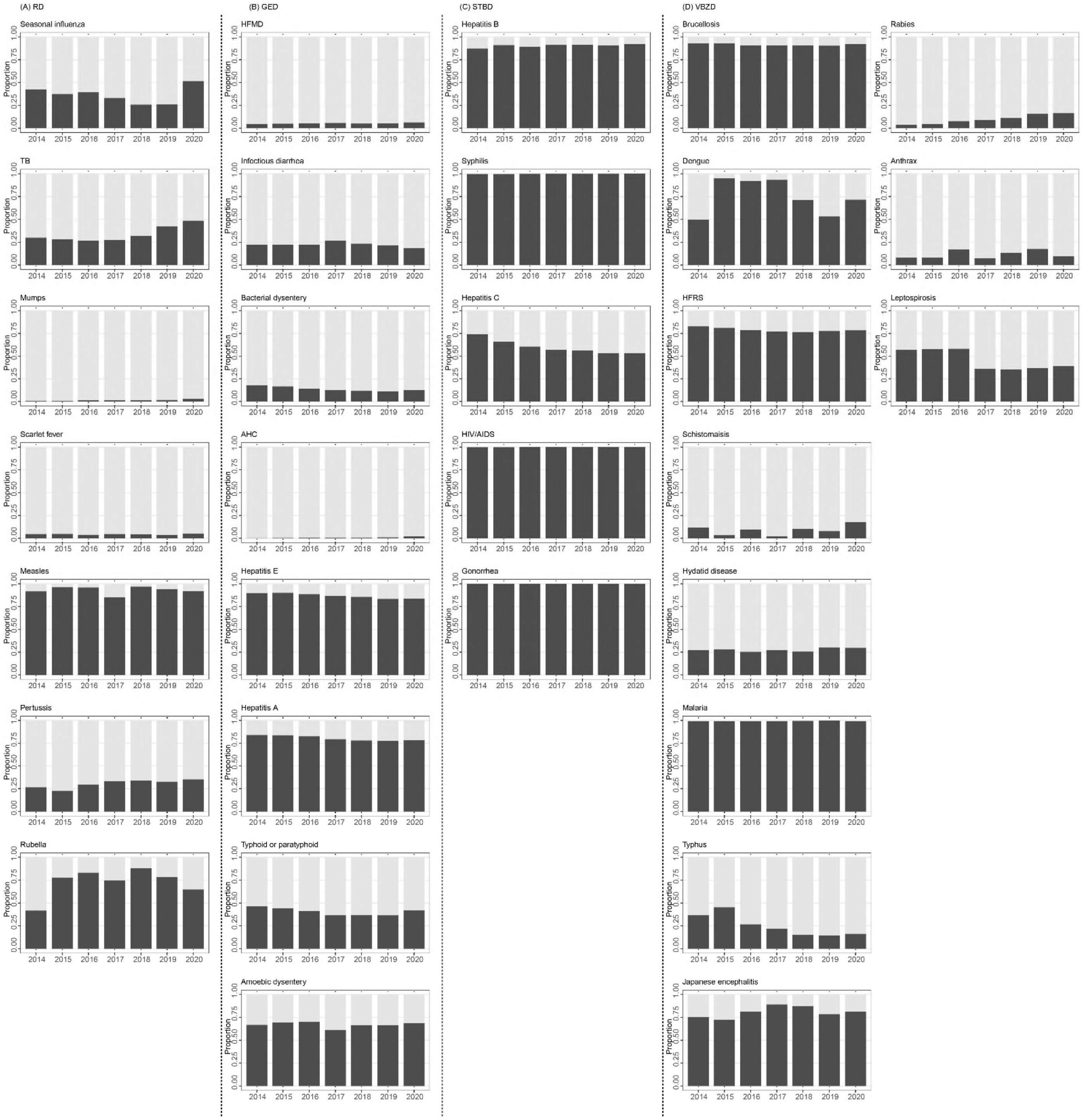

Supplementary Figure 3. The average proportions of lab confirmed cases among provinces in the mainland of China from 2014 to 2020. The sectors of each circle represent provinces, and the shades indicate the proportions of lab confirmed cases (dark) and clinically confirmed cases (light). The white (empty) sectors correspond to provinces where the numbers of reported cases are small (<50) and thus the proportions are not calculated. The proportion of lab confirmed cases varies from 0% to 100% from the center to the outer bound of each circle. Abbreviated (full) province names are: BJ (Beijing), TJ (Tianjin), HE (Hebei), IM (Inner Mongolia), SX (Shanxi), LN (Liaoning), JL (Jilin), HL (Heilongjiang), SH (Shanghai), JS (Jiangsu), ZJ (Zhejiang), AH (Anhui), FJ (Fujian), JX (Jiangxi), SD (Shandong); HA (Henan), HB (Hubei), HN (Hunan), GD (Guangdong), GX (Guangxi), HI (Hainan), CQ (Chongqing), SC (Sichuan), GZ (Guizhou), YN (Yunan), XZ (Tibet), SN (Shaanxi), GS (Gansu), QH (Qinghai), NX (Ningxia), XJ (Xinjiang).

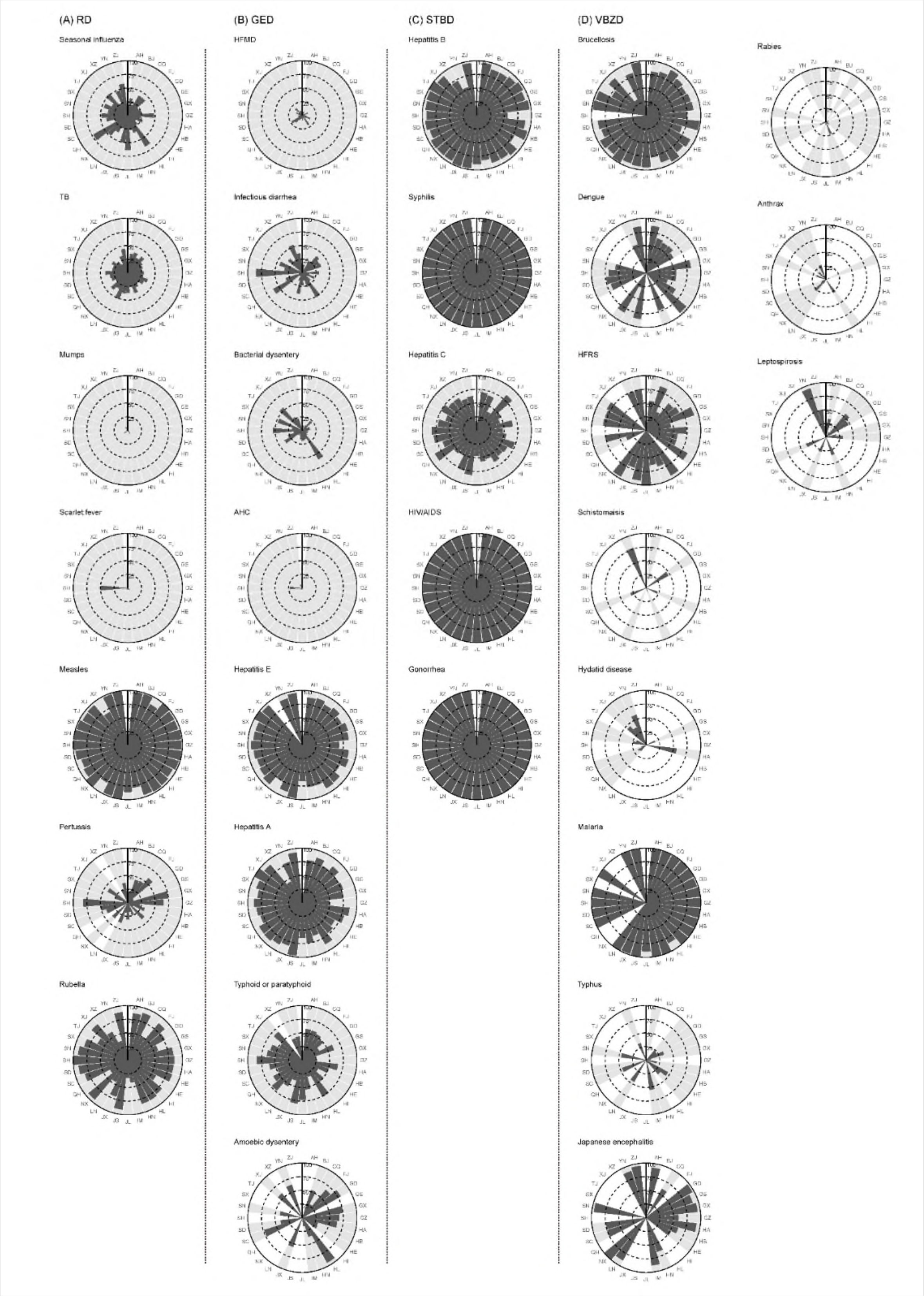

Supplementary Figure 4. Annual incidences of 31 notifiable infectious diseases from 2014 to 2020 in the mainland of China by age group, sex and region. Each concentric circle represents one year starting from 2014 (inner) to 2020 (outer). Each sector represents one infectious disease. Diseases were grouped into four categories (C1−C4): respiratory diseases, gastrointestinal or enteroviral diseases, sexually transmitted or bloodborne diseases, and vector-borne or zoonotic diseases. The colors of the largest outer ring indicate the average annual incidence of each category over 2014−2020.

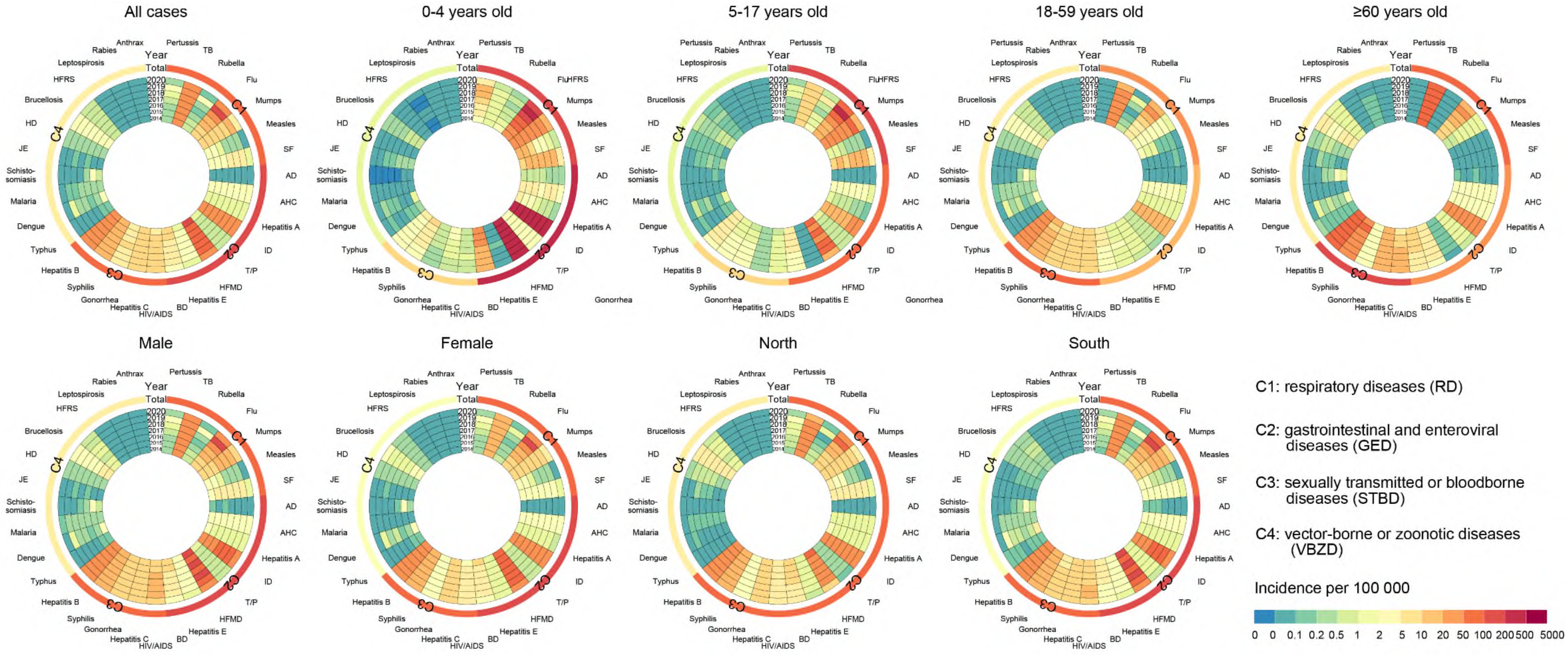

Supplementary Table 1. Comparation of annual incident case numbers and case fatality ratios between 2014–2019 (average) and 2020. Case fatality ratios in 2020 are colored red for increases and blue for decreases relative to 2014−2019.

Disease	Annual incident cases		Annual deaths		Case fatality ratio (‰, per thousand)		*p* value*
	2014-2019	2020	2014-2019	2020	2014-2019	2020	
**Respiratory diseases**	2161753	2037302	2930	2173	1.355	1.067	<0.001
Seasonal influenza	922816	1155247	101	86	0.109	0.074	0.001
Tuberculosis	889264	721903	2813	2084	3.163	2.887	<0.001
Mumps	232855	134741	0.2	1	0.001	0.007	0.168
Scarlet fever	70789	17480	0.2	1	0.002	0.057	0.077
Measles	22064	900	14	0	0.635	0.000	1.000
Pertussis	13459	4823	1.8	1	0.136	0.207	0.502
Rubella	10506	2208	0.2	0	0.016	0	1.000
**Gastrointestinal or enteroviral diseases**	3624282	1995969	207	41	0.057	0.021	<0.001
Hand, foot and mouth disease	2279045	792009	163	3	0.072	0.004	<0.001
Infectious diarrhea	1127521	1071993	18	13	0.016	0.012	0.439
Bacterial dysentery	116703	58817	4	4	0.031	0.068	0.138
Acute hemorrhagic conjunctivitis	38662	30259	0	0	0	0	1.000
Hepatitis E	28268	19243	6	13	0.560	0.676	0.634
Hepatitis A	20946	15129	6	3	0.278	0.198	0.794
Typhoid and paratyphoid	12044	7825	1	5	0.097	0.639	0.004
Amoebic dysentery	1093	694	0	0	0	0	1.000
**Sexually transmitted or bloodborne diseases**	2054368	1924355	30583	37877	14.887	19.683	<0.001
Hepatitis B	1040313	948638	389	501	0.374	0.528	<0.001
Syphilis	485826	490676	57	63	0.117	0.128	0.543
Hepatitis C	219365	195659	105	112	0.480	0.572	0.097
HIV/AIDS	190125	182142	30031	37201	157.951	204.242	<0.001
Gonorrhea	118740	107240	1	0	0	0	0.605
**Vector-borne or zoonotic diseases**	91750	63163	719	269	7.837	4.259	<0.001
Brucellosis	47175	47133	1	1	0.025	0.021	1.000
Dengue	14955	802	2	0	0.145	0.000	1.000
Hemorrhagic fever with renal syndrome	10734	8315	68	49	6.289	5.893	0.723
Schistosomiasis	7806	44	0	0	0	0	1.000
Hydatid disease	4386	3369	1	1	0.190	0.297	0.515
Malaria	2980	1058	16	6	5.257	5.671	1.000
Typhus	1293	1223	0.5	0	0.387	0	1.000
Japanese encephalitis	1083	323	56	10	51.578	30.960	0.147
Leptospirosis	338	377	2	8	6.404	21.220	0.011
Rabies	621	216	571	194	919.216	898.148	0.859
Anthrax	378.8	303	2	0	6.159	0	0.394
**Total**	7932153	6020789	34439	40360	4.342	6.703	<0.001

* Two-sided *p*-values were calculated by Pearson Chi-square test and Fisher's exact test (for sparse counts).

Supplementary Figure 5. Cumulative incidences of selected notifiable infectious disease across four COVID-19 intervention phases in 2020 (red), compared to the average cumulative incidences during 2014 – 2019 in the corresponding time intervals (blue). (A) Respiratory disease; (B) Gastrointestinal or enteroviral disease; (C) Sexually transmitted or bloodborne disease; (D) Vector-borne or zoonotic disease. The four phases are defined as Jan 1–22 (Phase I), Jan 23–Apr 7 (Phase II), Apr 8–Aug 31 (Phase III) and Sep 1–Dec 31 (Phase IV). "Others" in panel D combines rabies, anthrax and leptospirosis.

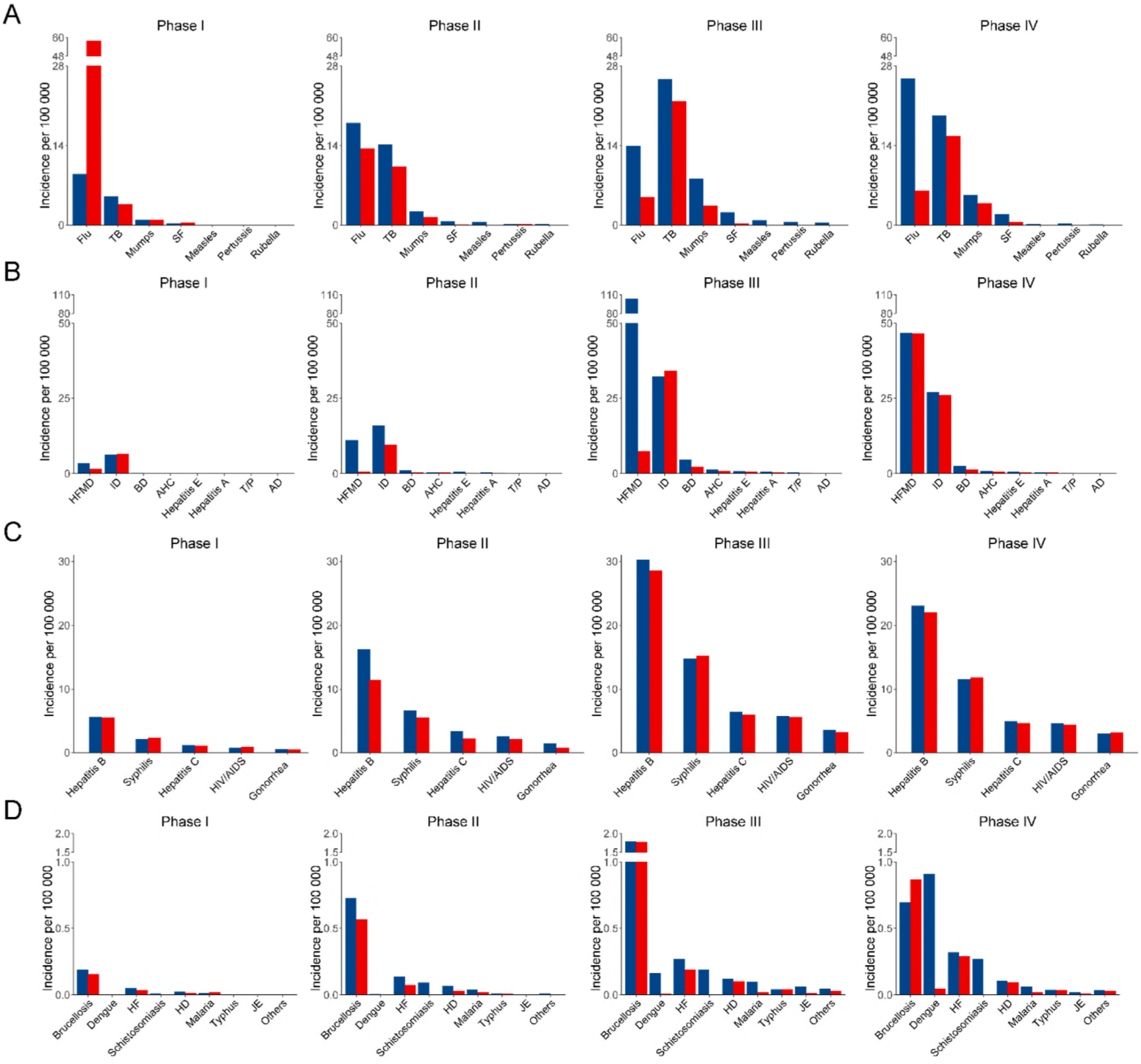

Supplementary Figure 6. Percent change of province-level incidences of selected respiratory diseases in four COVID-19 intervention phases of 2020, compared to the average incidences during 2014－2019 in corresponding time intervals. The four phases are defined as Jan 1–22 (Phase I), Jan 23–Apr 7 (Phase II), Apr 8–Aug 31 (Phase III) and Sep 1–Dec 31 (Phase IV). The gradient red (blue) colors indicate higher (lower) incidences in 2020 than the average during 2014－2019. Gray indicates missing value.

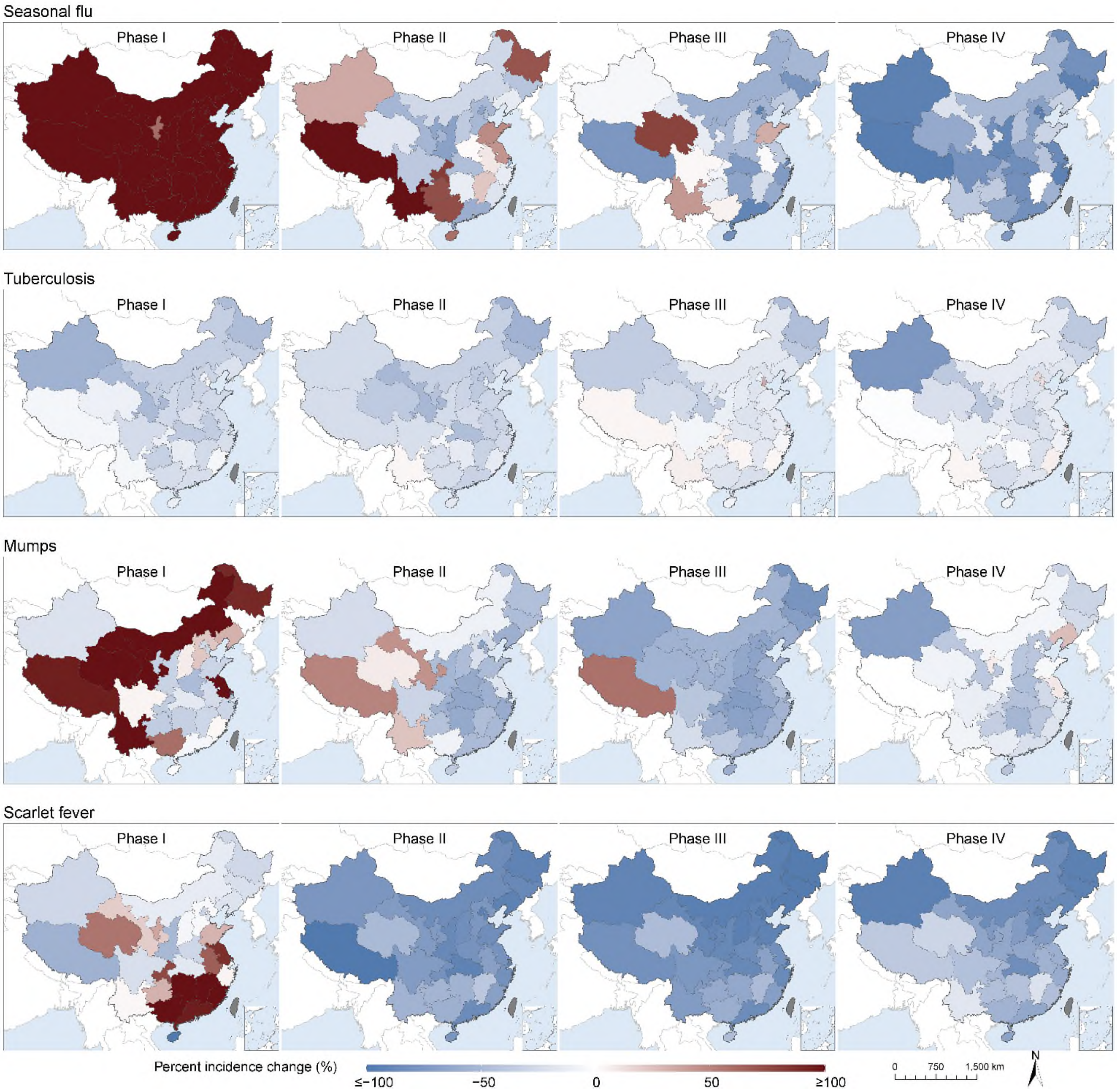

Supplementary Figure 6 continued.

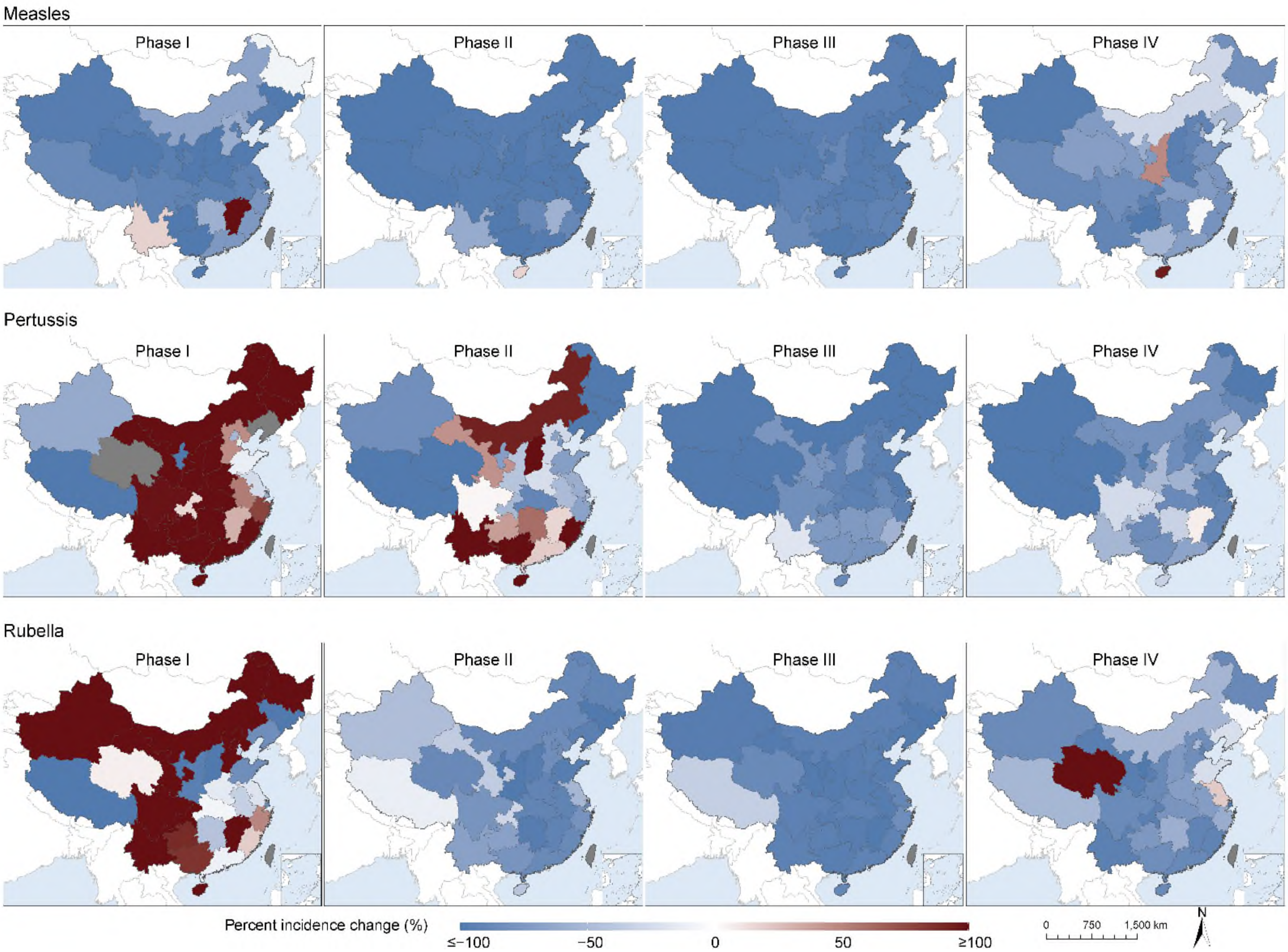

Supplementary Figure 7. Percent changes of incidences of selected respiratory diseases in four COVID-19 intervention phases of 2020 compared to the average incidences during 2014 – 2019 in corresponding time intervals, stratified by age group and sex. Red and blue bars indicate positive and negative percent changes, respectively. (A) <5 years old; (B) 5−17 years old; (C) 18−59 years old; (D) ≥60 years old; (E) Males (solid bars) vs. females (unfilled bars). The black lines indicate the 95% CIs of the percent changes. The percent change was calculated by: $[(inc_{2020}(k) - inc_{2014-2019}(k)]/inc_{2014-2019}(k) \times 100\%$, where $inc_{2014-2019}(k)$ indicates the average incidence during phase k over 2014 to 2019 and $inc_{2020}(k)$ indicates the incidence specific to phase k of 2020 as shown in Table 1 in the main text. The black lines indicate the error bars. The error bars indicating 95% confidence intervals for the percent changes were calculated by the delta method.

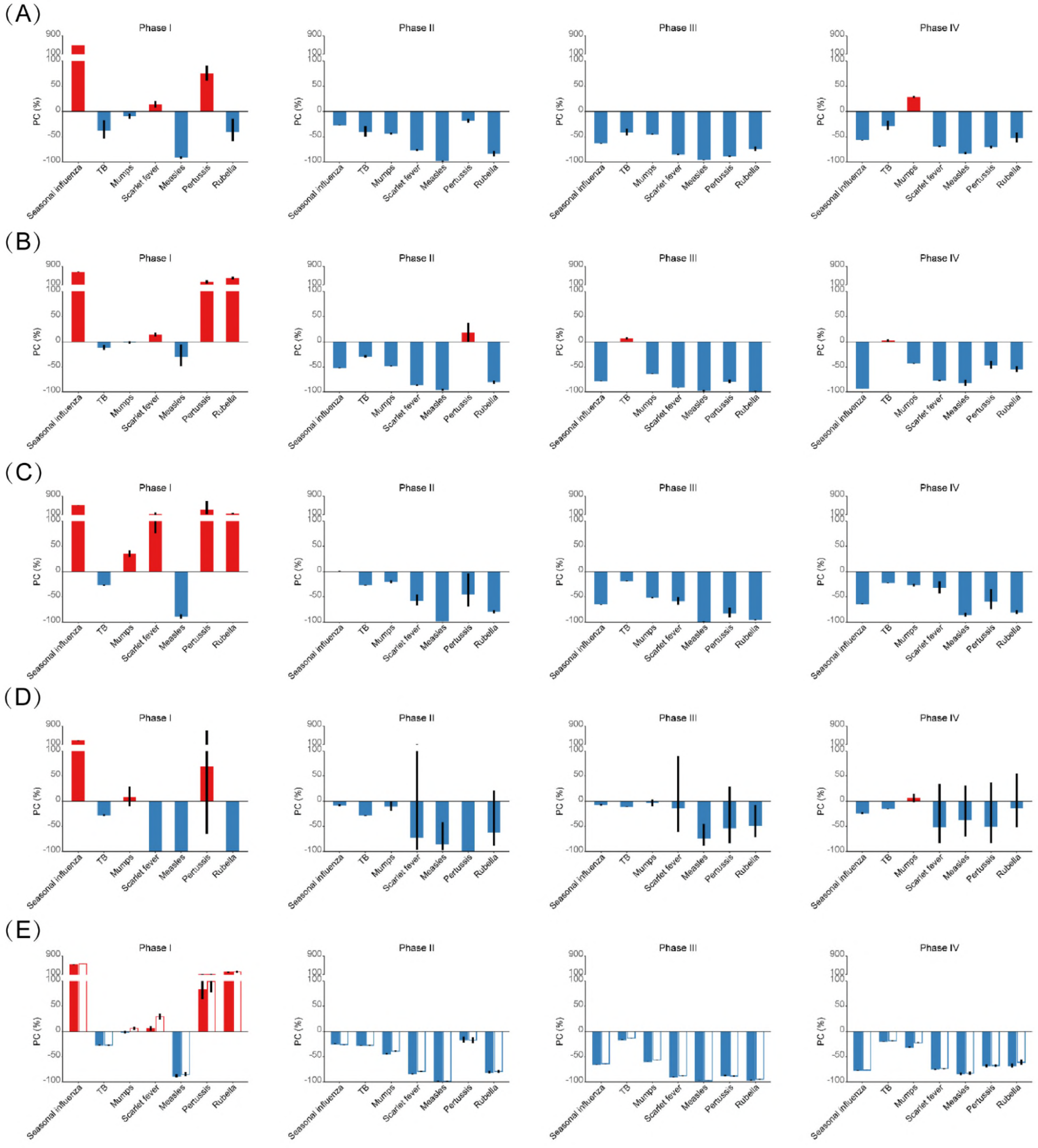

Supplementary Figure 8. Percent changes of province-level incidences of selected gastrointestinal or enteroviral diseases in four COVID-19 intervention phases of 2020, compared to the average incidences during 2014 – 2019 in corresponding time intervals. The four phases are defined as Jan 1–22 (Phase I), Jan 23–Apr 7 (Phase II), Apr 8–Aug 31 (Phase III) and Sep 1–Dec 31 (Phase IV). The gradient red (blue) colors indicate higher (lower) incidences in 2020 than the average during 2014–2019. Gray indicates missing value.

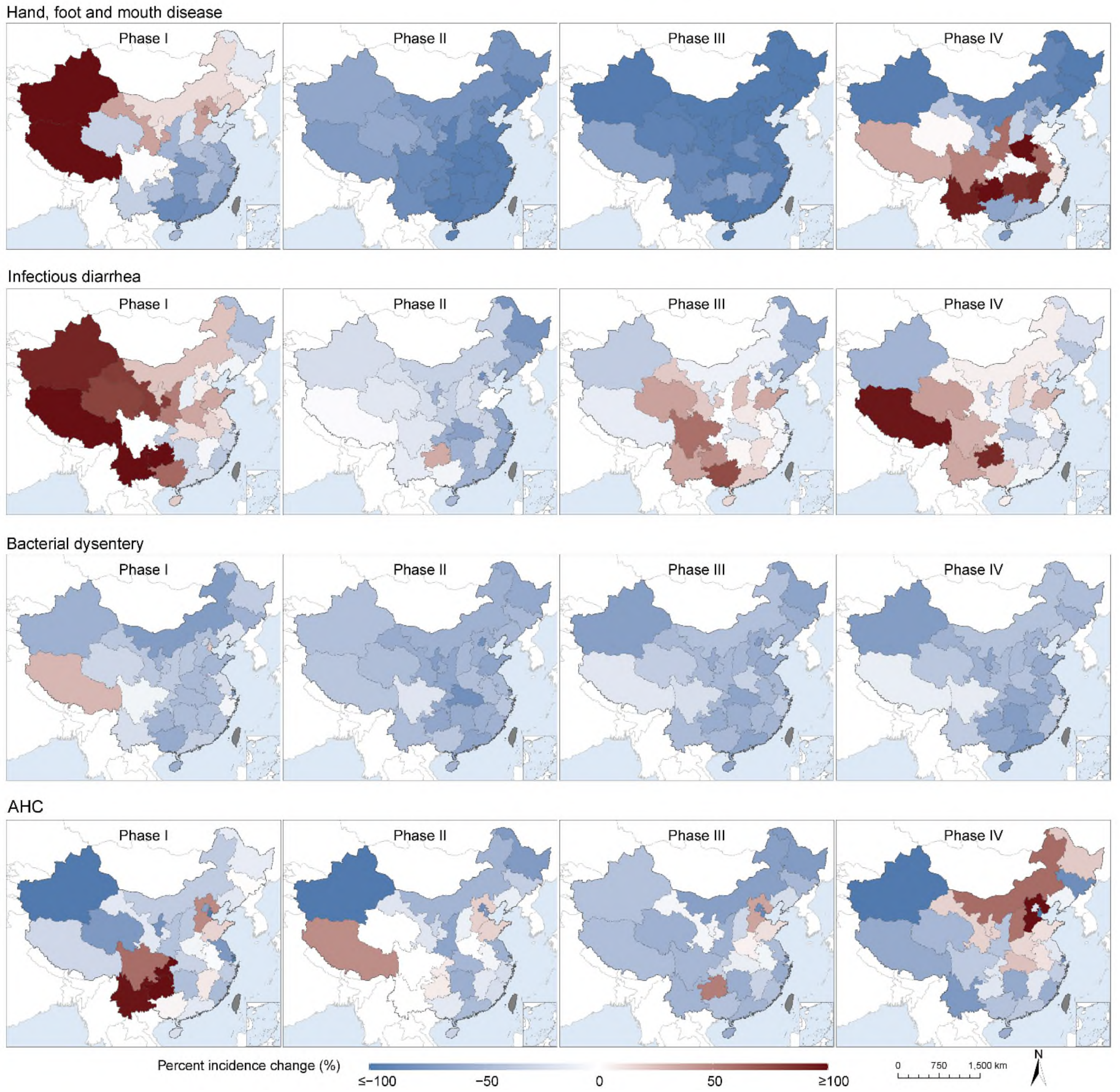

Supplementary Figure 8 continued.

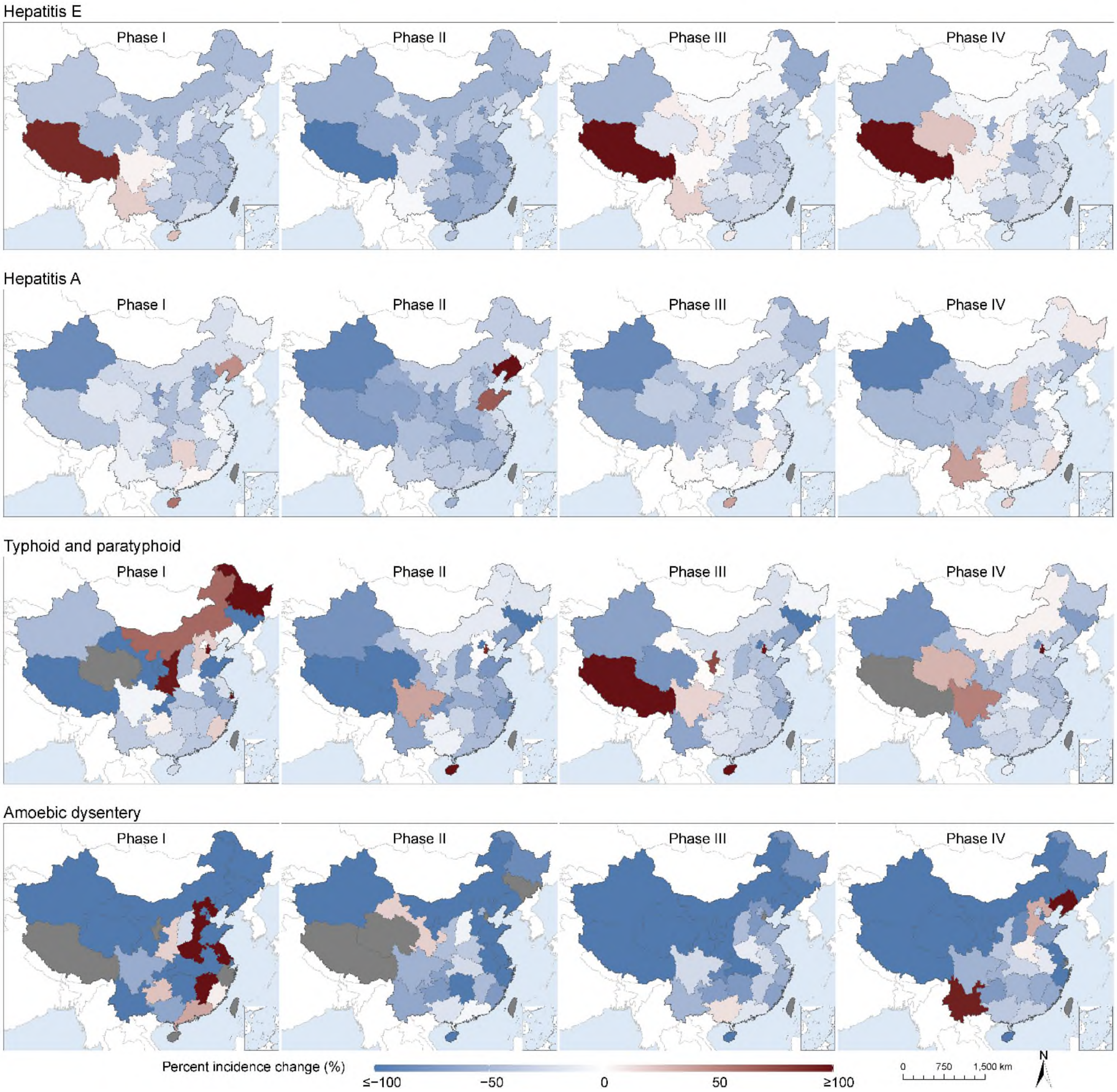

Supplementary Figure 9. Percent changes of incidences of selected gastrointestinal or enteroviral diseases in four COVID-19 intervention phases of 2020 compared to the average incidences during 2014−2019 in corresponding time intervals, stratified by age group and sex. Red and blue bars indicate positive and negative percent changes, respectively. (A) <5 years old; (B) 5–17 years old; (C) 18–59 years old; (D) ≥60 years old; (E) Males (solid bars) vs. females (unfilled bars). The black lines indicate the 95% CIs of the percent changes. The percent change was calculated by: $[(inc_{2020}(k) - inc_{2014-2019}(k)]/inc_{2014-2019}(k) \times 100\%$, where $inc_{2014-2019}(k)$ indicates the average incidence during phase k over 2014 to 2019 and $inc_{2020}(k)$ indicates the incidence specific to phase k of 2020 as shown in Table 1 in the main text. The black lines indicate the error bars. The error bars indicating 95% confidence intervals for the percent changes were calculated by the delta method.

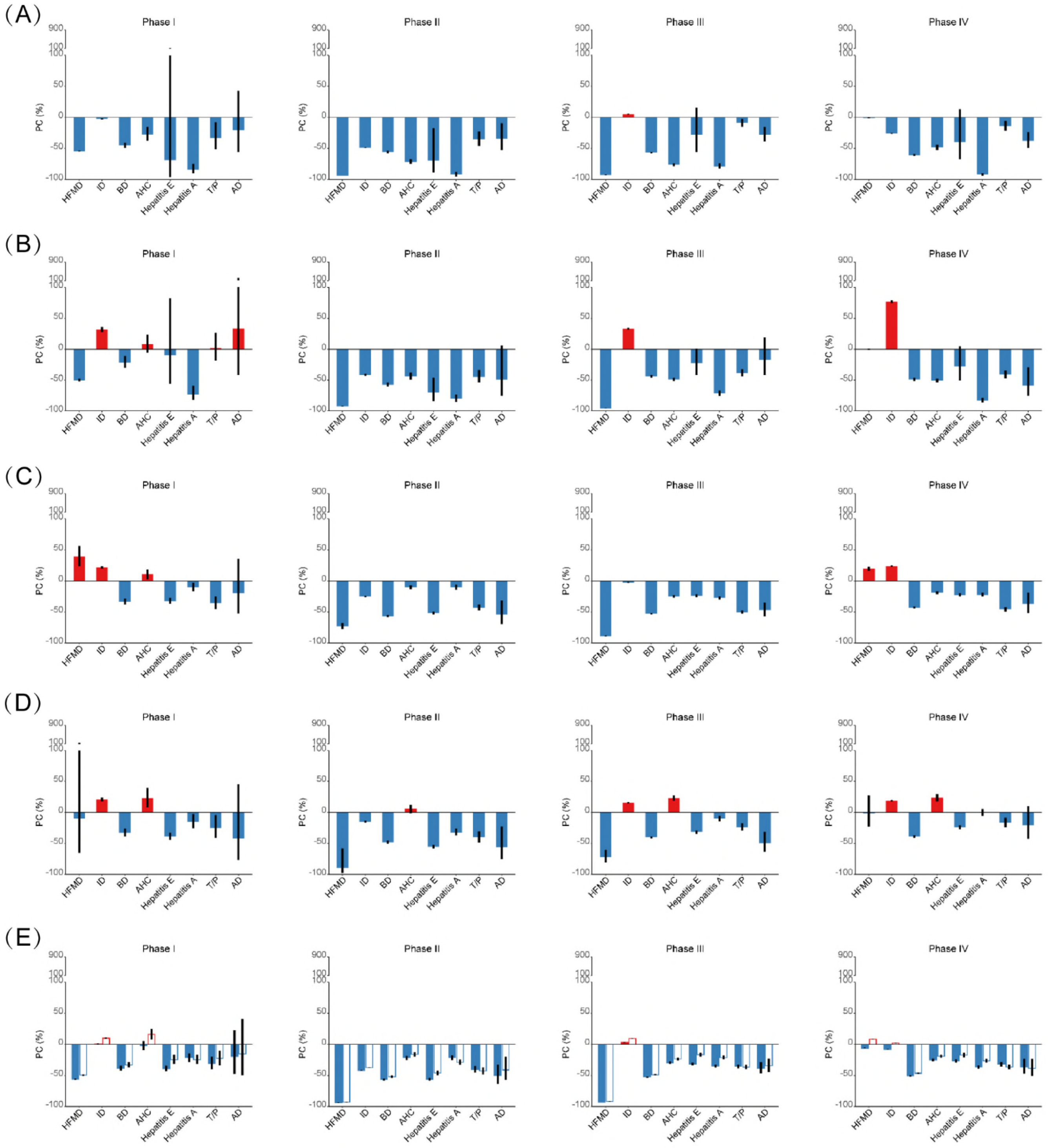

Supplementary Figure 10. Percent changes of province-level incidences of selected sexually transmitted or bloodborne diseases in four COVID-19 intervention phases of 2020, compared to the average incidences during 2014 – 2019 in corresponding time intervals. The four phases are defined as Jan 1–22 (Phase I), Jan 23–Apr 7 (Phase II), Apr 8–Aug 31 (Phase III) and Sep 1–Dec 31 (Phase IV). The gradient red (blue) colors indicate higher (lower) incidences in 2020 than the average during 2014–2019. Gray indicates missing value.

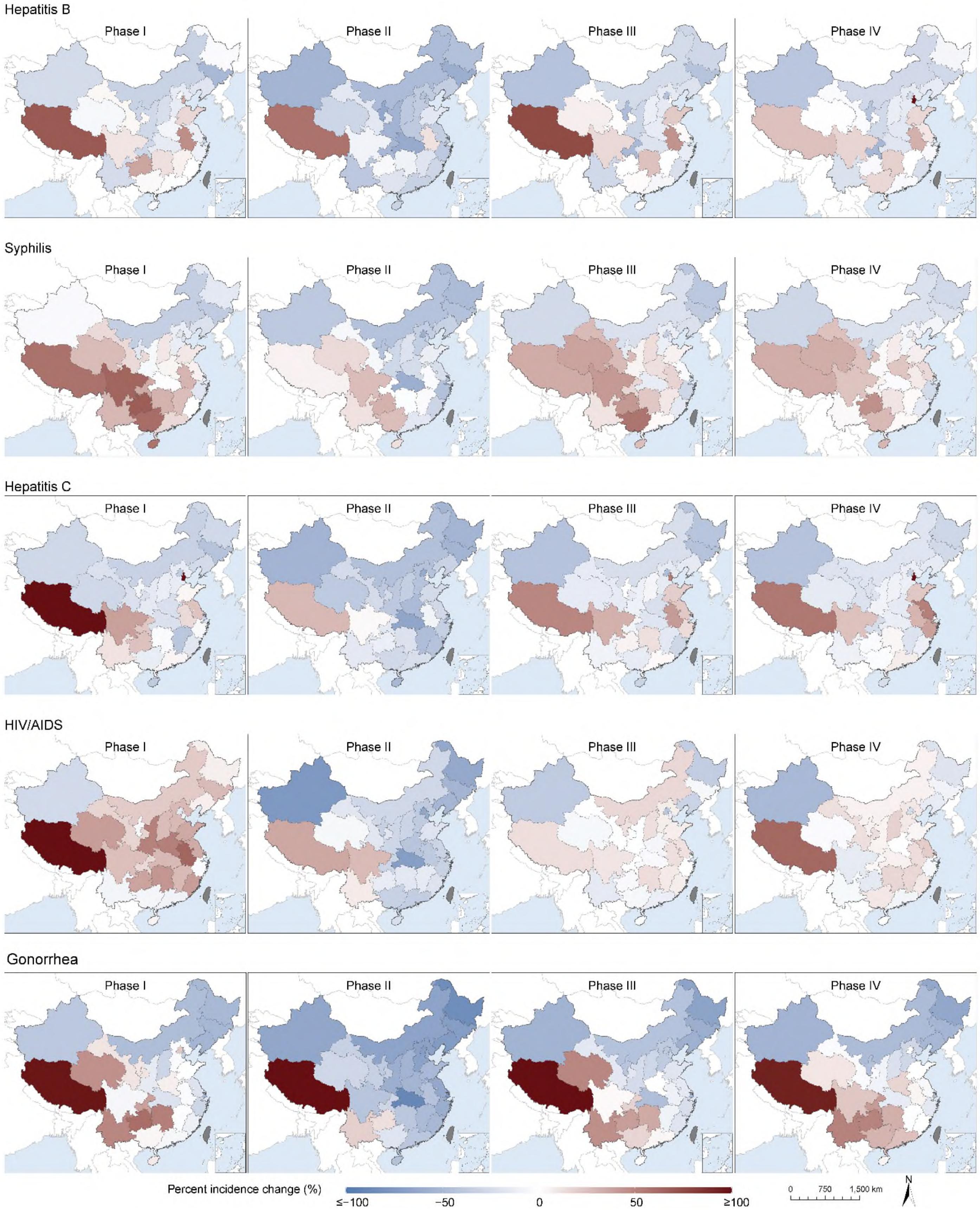

Supplementary Figure 11. Percent changes of incidences of selected sexually transmitted or bloodborne diseases in four COVID-19 intervention phases of 2020 compared to the average incidences during 2014 – 2019 in corresponding time intervals, stratified by age group and sex. Red and blue bars indicate positive and negative percent changes, respectively. (A) <5 years old; (B) 5–17 years old; (C) 18–59 years old; (D) ≥60 years old; (E) Males (solid bars) vs. females (unfilled bars). The black lines indicate the 95% CIs of the percent changes. The percent change was calculated by: $[(inc_{2020}(k) - inc_{2014-2019}(k)]/inc_{2014-2019}(k) \times 100\%$, where $inc_{2014-2019}(k)$ indicates the average incidence during phase k over 2014 to 2019 and $inc_{2020}(k)$ indicates the incidence specific to phase k of 2020 as shown in Table 1 in the main text. The black lines indicate the error bars. The error bars indicating 95% confidence intervals for the percent changes were calculated by the delta method.

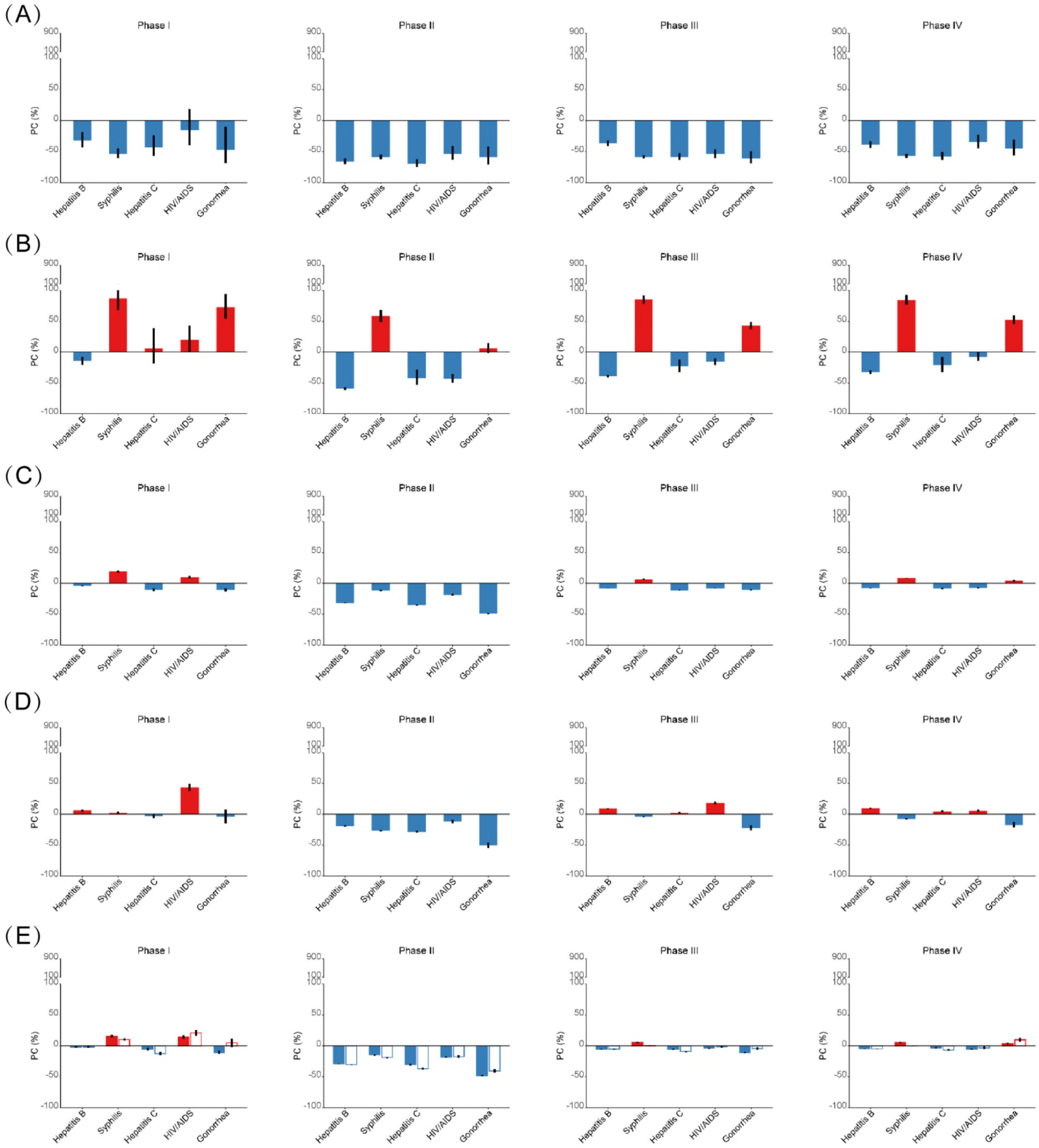

Supplementary Figure 12. Percent changes of province-level incidences of selected vector-borne or zoonotic diseases in four COVID-19 intervention phases of 2020, compared to the average incidences during 2014 – 2019 in corresponding time intervals. The four phases are defined as Jan 1–22 (Phase I), Jan 23–Apr 7 (Phase II), Apr 8–Aug 31 (Phase III) and Sep 1–Dec 31 (Phase IV). The gradient red (blue) colors indicate higher (lower) incidences in 2020 than the average during 2014–2019. Gray indicates missing value.

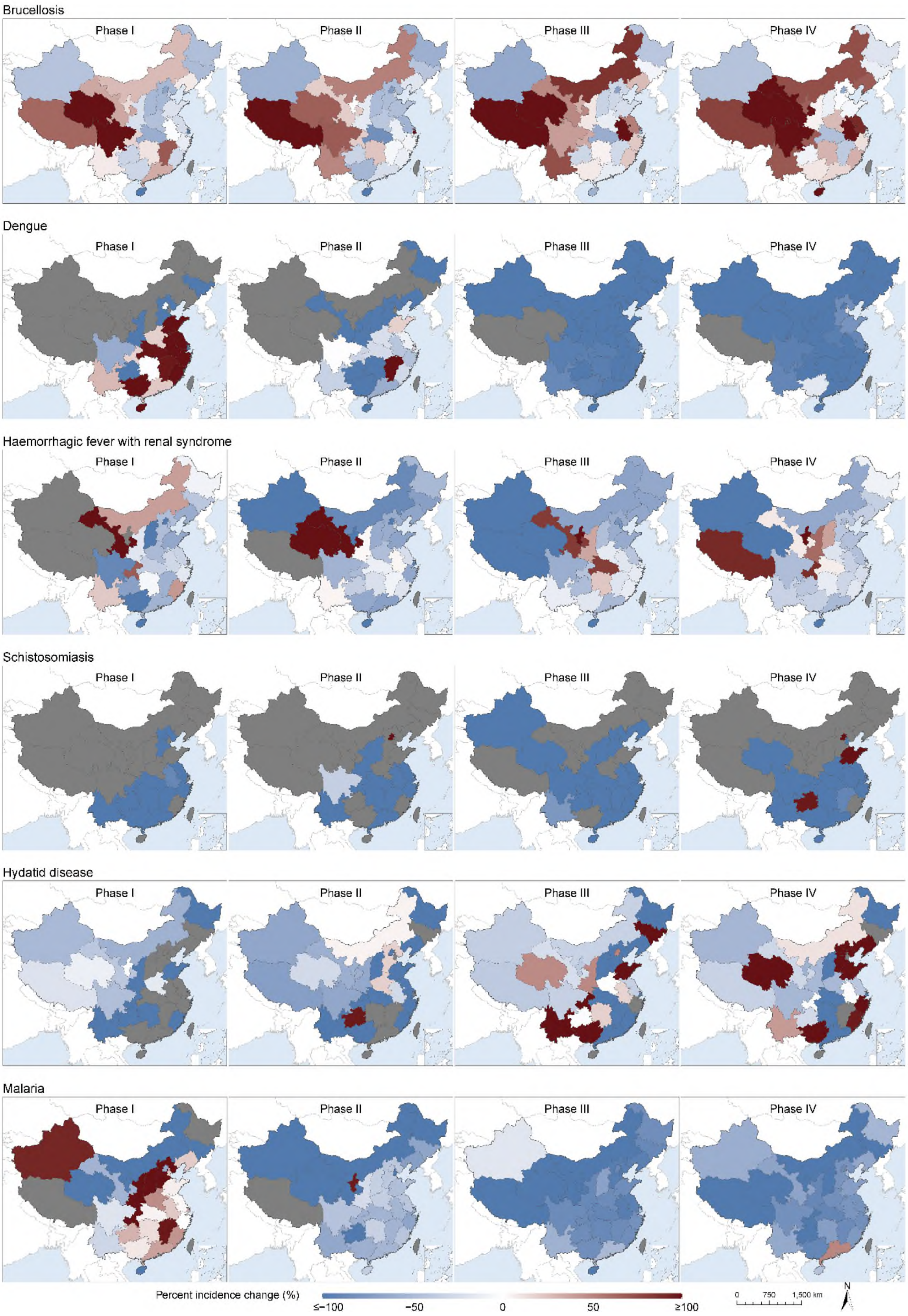

Supplementary Figure 12 continued.

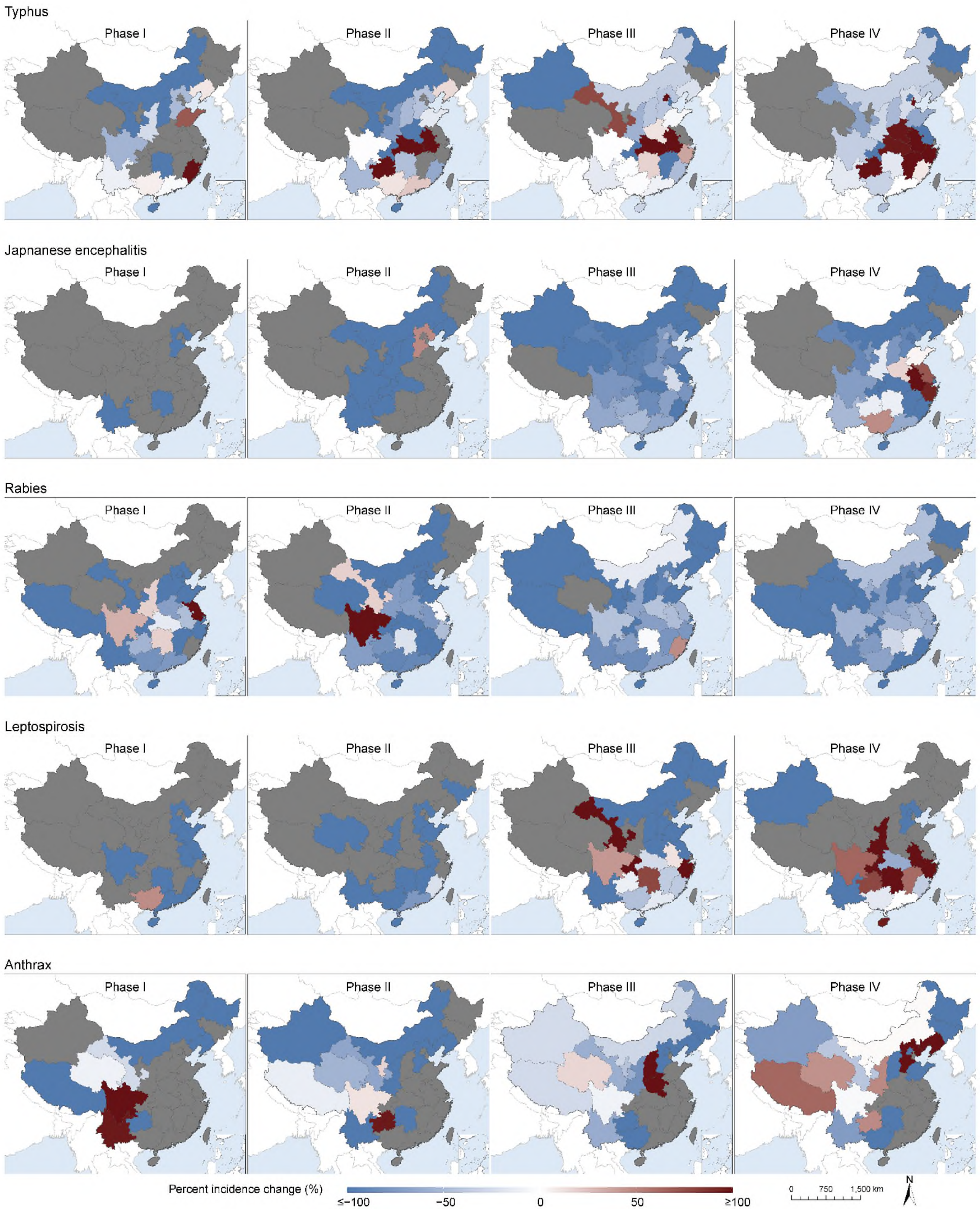

Supplementary Figure 13. Percent changes of incidences of selected vector-borne or zoonotic diseases in four COVID-19 intervention phases of 2020 compared to the average incidences during 2014–2019 in corresponding time intervals, stratified by age group and sex. Red and blue bars indicate positive and negative percent changes, respectively. (A) <5 years old; (B) 5–17 years old; (C) 18–59 years old; (D) ≥60 years old; (E) Males (solid bars) vs. females (unfilled bars). The percent change was calculated by: $[(inc_{2020}(k) - inc_{2014-2019}(k))]/inc_{2014-2019}(k) \times 100\%$, where $inc_{2014-2019}(k)$ indicates the average incidence during phase *k* over 2014 to 2019 and $inc_{2020}(k)$ indicates the incidence specific to phase *k* of 2020 as shown in Table 1 in the main text. The black lines indicate the error bars. The error bars indicating 95% confidence intervals for the percent changes were calculated by the delta method.

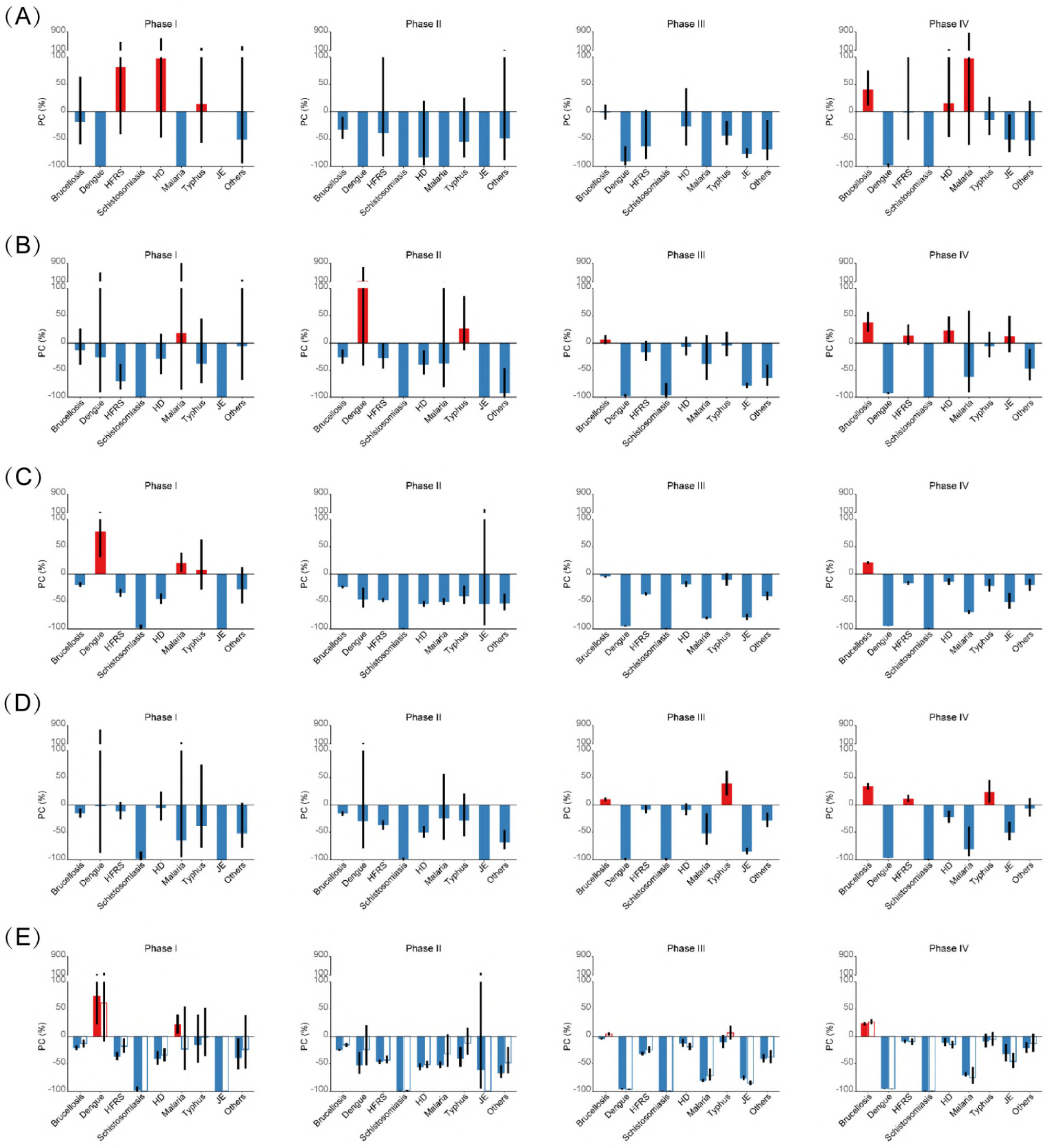

Supplementary Figure 14. Comparison of annual incidences of lab confirmed cases between 2014−2019 (average, blue) and 2020 (red) for selected nine infectious diseases. Comparison is stratified by time intervals corresponding to the four COVID-19 intervention phases during 2020.

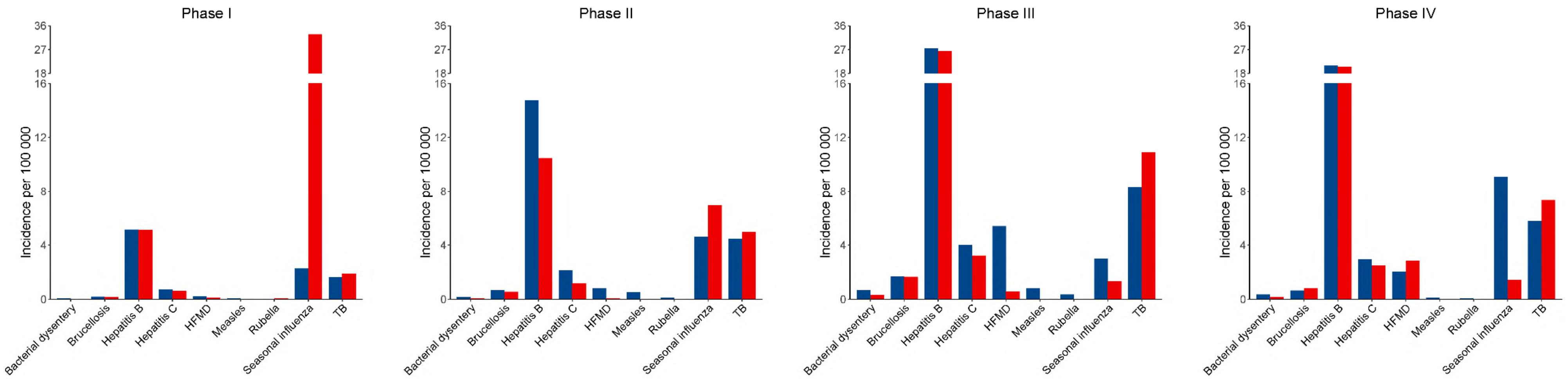

Supplementary Figure 15. Relative changes of monthly number of reported cases and volume of outpatient visits from 2019 to 2020 for each category of notifiable diseases in South and North China.

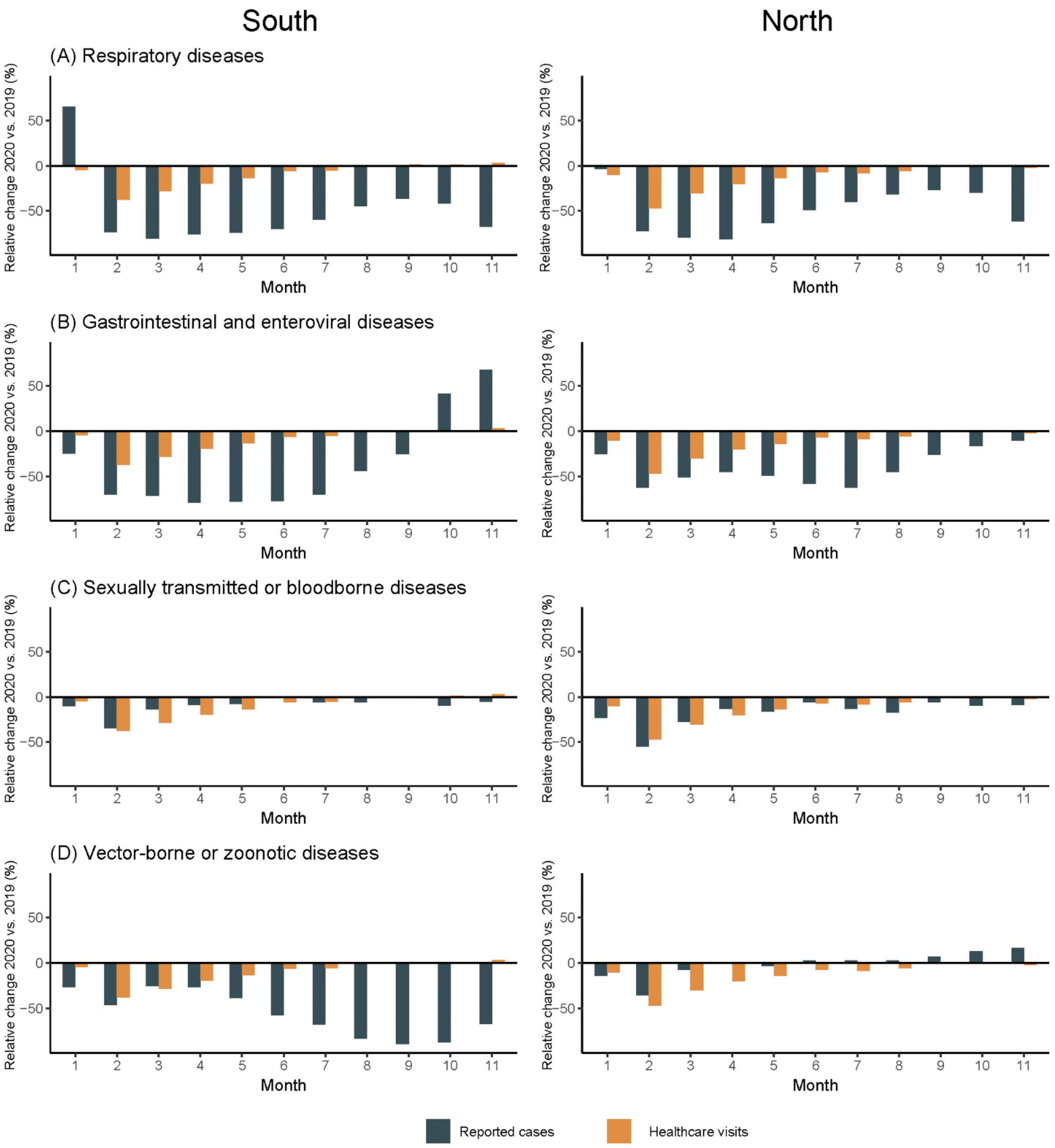

Supplementary Figure 16. Time series of observed (black dots) and GLM-projected monthly numbers of reported cases for selected sexually transmitted or bloodborne diseases. (A) Gonorrhea; (B) Syphilis; (C) HIV/AIDS; (D) Hepatitis B; (E) Hepatitis C. The model-projected trajectories are shown for both with (black solid) and without (blue dash, 2020 only) nonpharmaceutical interventions. Intervention phases II−IV (Feb to Dec) in 2020 are colored light blue. For the counterfactual trajectory without NPIs, monthly volumes of outpatient visits in 2020 were assumed the same as in 2019.

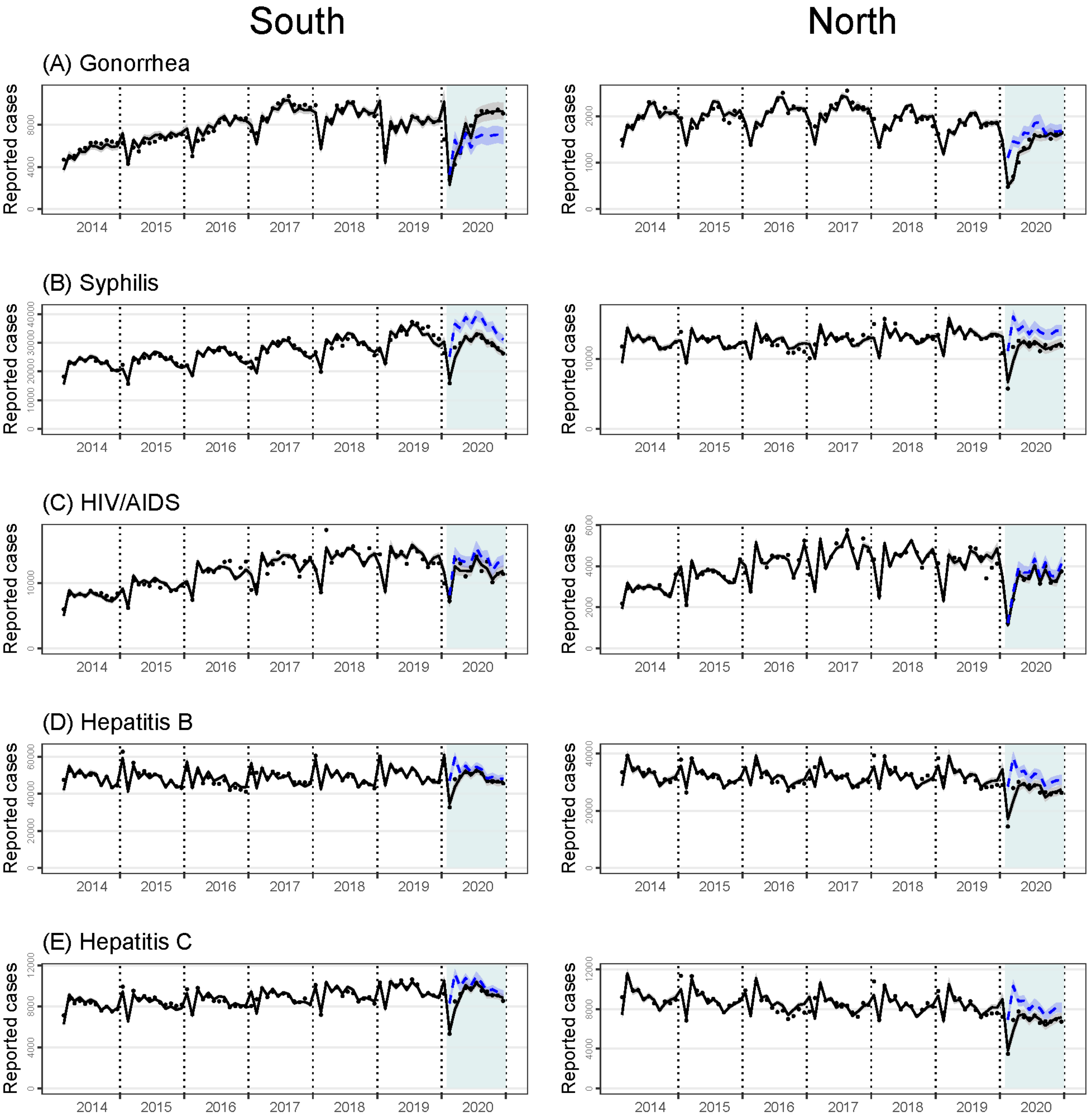

Supplementary Figure 17. Time series of observed (black dots) and GLM-projected monthly numbers of reported cases for selected vector-borne or zoonotic diseases. (A) Brucellosis; (B) HFRS; (C) Malaria; (D) Hydatid disease; (E) Dengue; (F) Typhus. The model-projected trajectories are shown for both with (black solid) and without (blue dash, 2020 only) nonpharmaceutical interventions. Intervention phases II−IV (Feb to Dec) in 2020 are colored light blue. For the counterfactual trajectory without NPIs, monthly volumes of outpatient visits in 2020 were assumed the same as in 2019.

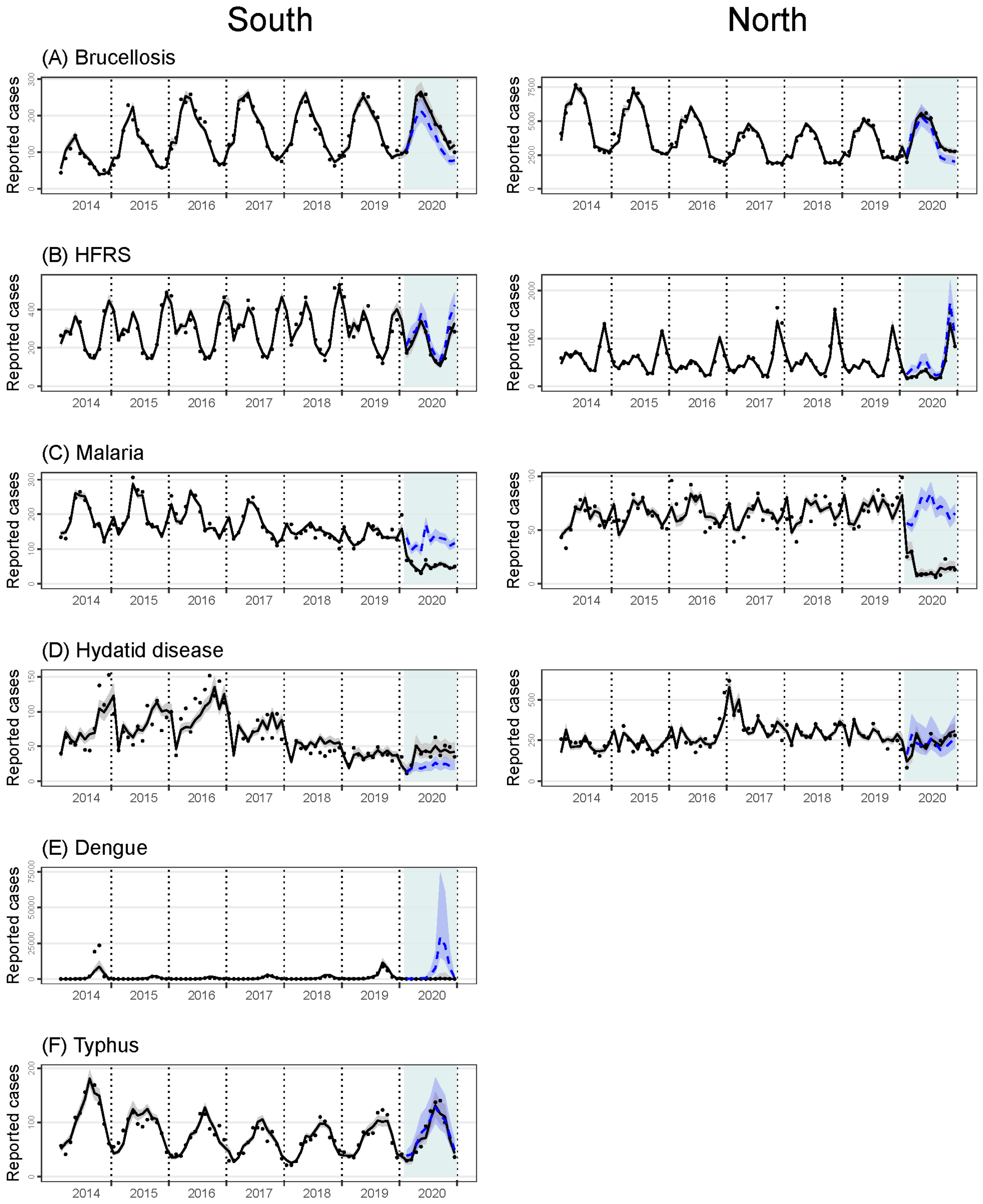

Supplementary Figure 18. Time series of observed (black dots) and GLM-projected monthly numbers of reported cases for selected respiratory diseases in the sensitivity analysis where harmonic functions instead of SEATS were used to adjust for seasonality. (A) Measles; (B) Mumps; (C) Pertussis; (D) Rubella; (E) Scarlet fever; (F) Seasonal influenza; (G) Tuberculosis. The model-projected trajectories are shown for both with (black solid) and without (blue dash, 2020 only) nonpharmaceutical interventions. Intervention phases II−IV (Feb to Dec) in 2020 are colored light blue. For the counterfactual trajectory without NPIs, monthly volumes of outpatient visits in 2020 were assumed the same as in 2019.

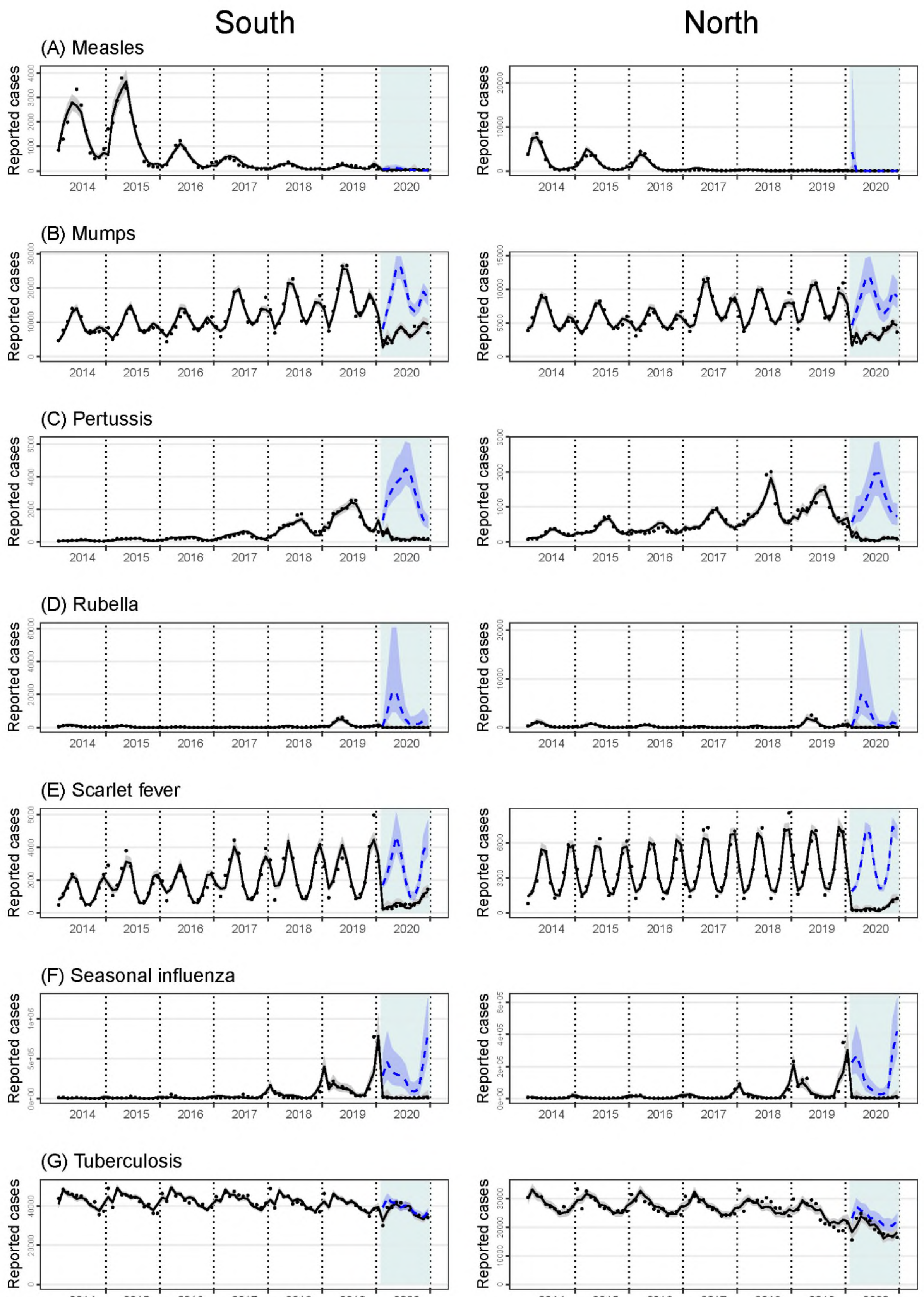

Supplementary Figure 19. Time series of observed (black dots) and GLM-projected monthly numbers of reported

cases for selected gastrointestinal or enteroviral diseases in the sensitivity analysis where harmonic functions instead of SEATS were used to adjust for seasonality. (A) Acute hemorrhagic conjunctivitis; (B) Bacterial dysentery; (C) Hepatitis A; (D) Hepatitis E; (E) Hand, foot and mouth disease; (F) Infectious diarrhea; (G) Typhoid or paratyphoid; (H) Amoebic dysentery. The model-projected trajectories are shown for both with (black solid) and without (blue dash, 2020 only) nonpharmaceutical interventions. Intervention phases II−IV (Feb to Dec) in 2020 are colored light blue. For the counterfactual trajectory without NPIs, monthly volumes of outpatient visits in 2020 were assumed the same as in 2019.

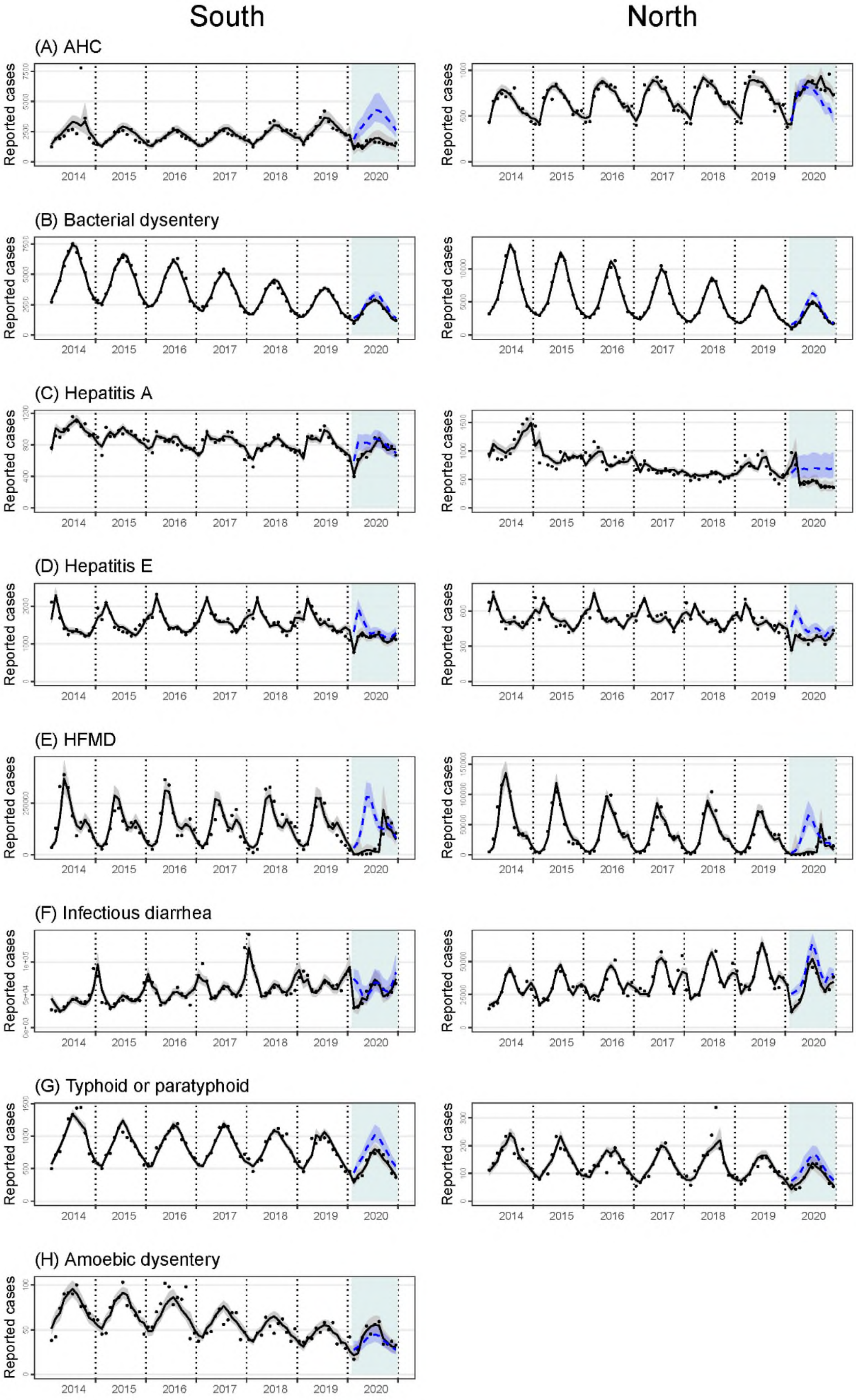

Supplementary Figure 20. Time series of observed (black dots) and GLM-projected monthly numbers of reported

cases for selected sexually transmitted or bloodborne diseases in the sensitivity analysis where harmonic functions instead of SEATS were used to adjust for seasonality. (A) Gonorrhea; (B) Syphilis; (C) HIV/AIDS; (D) Hepatitis B; (E) Hepatitis C. The model-projected trajectories are shown for both with (black solid) and without (blue dash, 2020 only) nonpharmaceutical interventions. Intervention phases II−IV (Feb to Dec) in 2020 are colored light blue. For the counterfactual trajectory without NPIs, monthly volumes of outpatient visits in 2020 were assumed the same as in 2019.

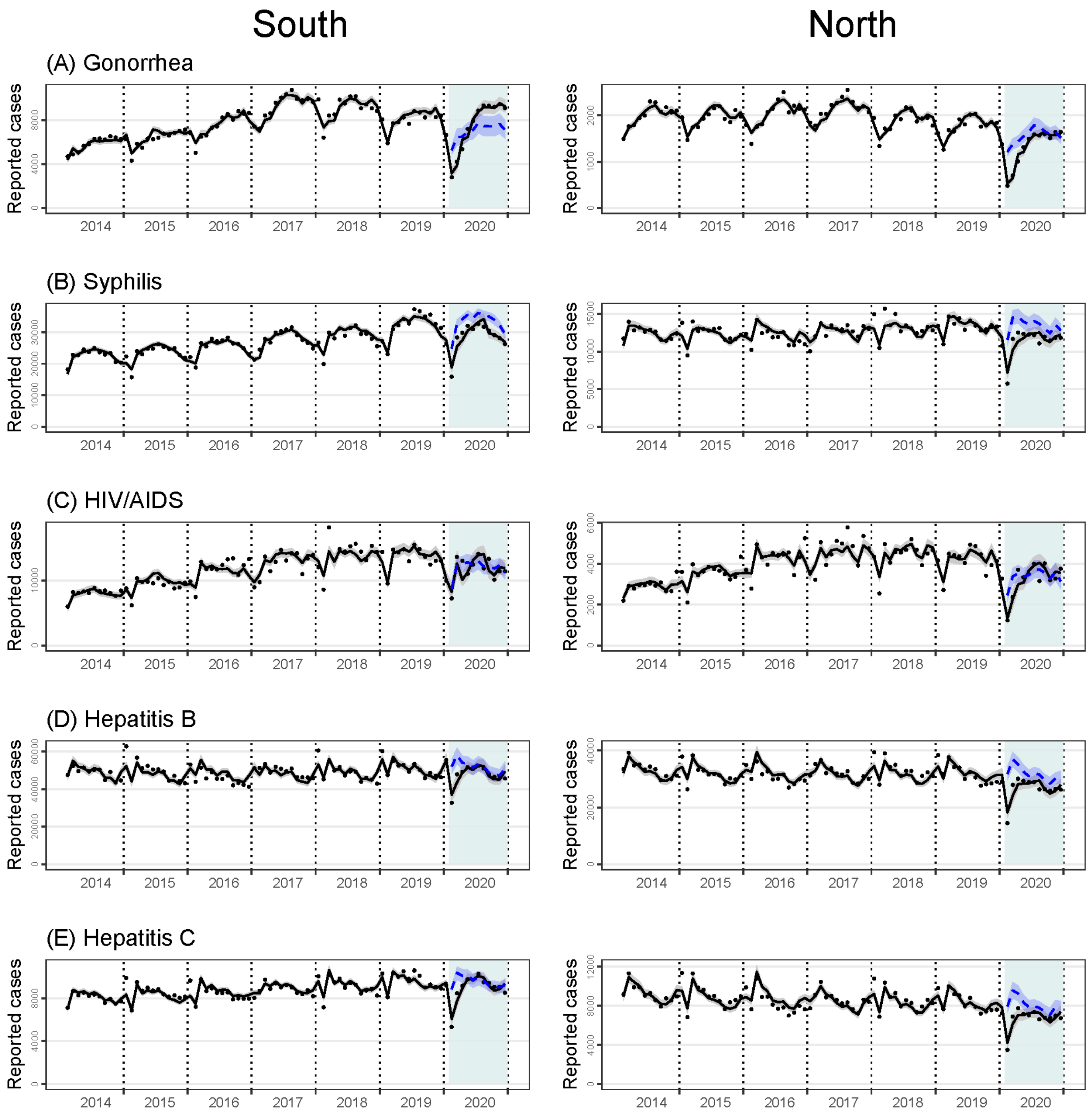

Supplementary Figure 21. Time series of observed (black dots) and GLM-projected monthly numbers of reported cases for selected vector-borne or zoonotic diseases in the sensitivity analysis where harmonic functions instead of SEATS were used to adjust for seasonality. (A) Brucellosis, (B) HFRS, (C) Malaria, (D) Hydatid disease, (E) Dengue, (F) Typhus. The model-projected trajectories are shown for both with (black solid) and without (blue dash, 2020 only) nonpharmaceutical interventions. Intervention phases II−IV (Feb to Dec) in 2020 are colored light blue. For the counterfactual trajectory without NPIs, monthly volumes of outpatient visits in 2020 were assumed the same as in 2019.

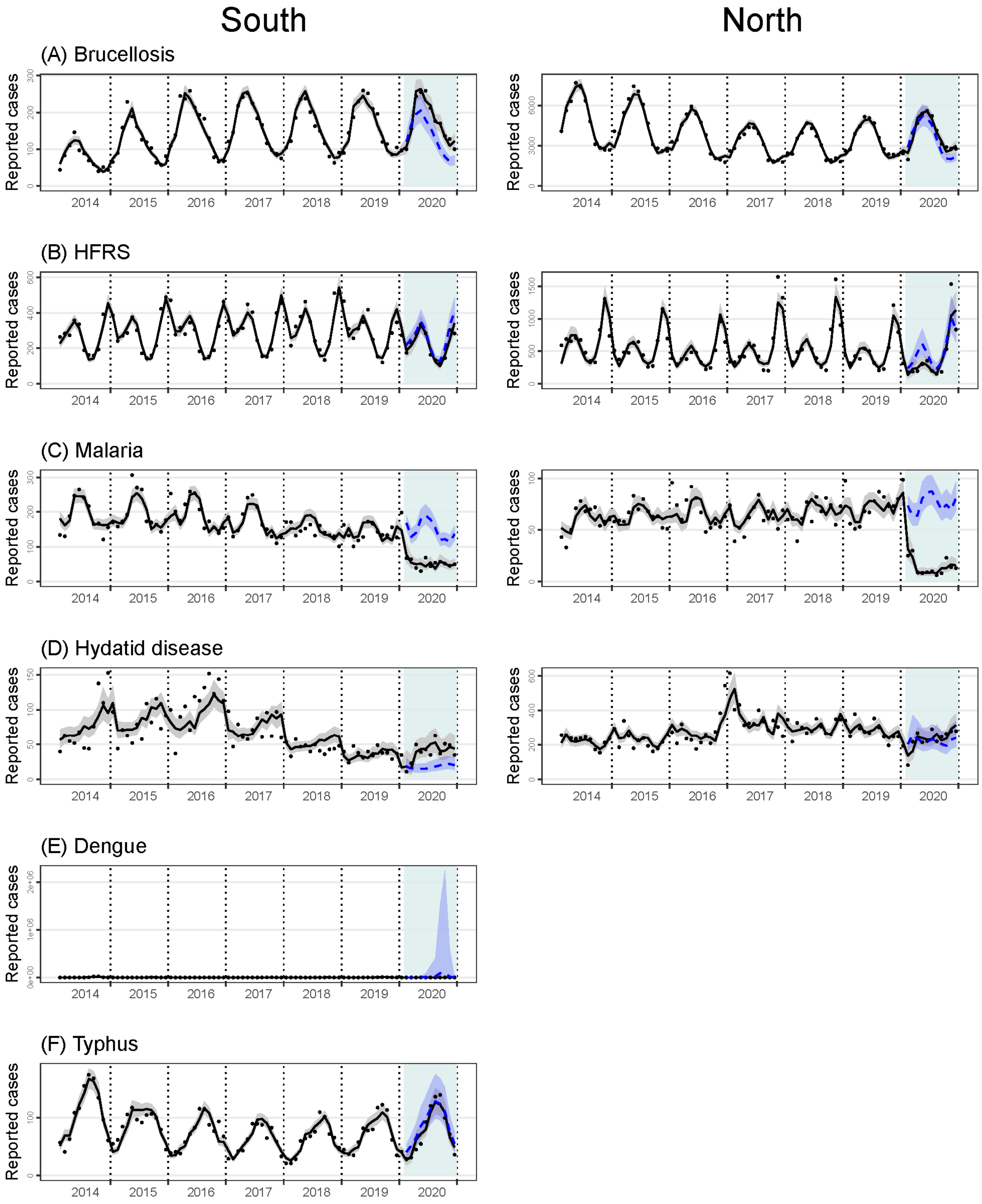

Supplementary Table 2. **Sensitivity analysis for model–estimated incidence rate ratio (IRR) of 26 selected infectious diseases.** Generalized linear models (GLM) were used for estimating the IRRs measuring the association of NPIs with disease trends. The models were adjusted for monthly healthcare visits, seasonality and yearly long-term trend, and Seasonality was adjusted for by harmonic functions in the GLM instead of SEATS. IRR bellow 1 with P<0.05 indicates significant decline of incidence rate in year 2020 compared to year 2014–2019. All p-values are two-sided and not adjusted for multiple comparisons.

Disease[‡]	South						North					
	Phase II[†]		Phase III[†]		Phase IV[†]		Phase II[†]		Phase III[†]		Phase IV[†]	
	IRR (95%CI)	p value	IRR (95%CI)	p value	IRR (95%CI)	p value	IRR (95%CI)	p value	IRR (95%CI)	p value	IRR (95%CI)	p value
**Respiratory diseases**												
Measles	0.44 (0.06–3.23)	0.424	0.58 (0.20–1.66)	0.313	0.99 (0.34–2.90)	0.987	**0.01 (0.0002–0.91)**	**0.049**	4.17 (0.43–40.15)	0.220	**10.24 (1.05–99.63)**	**0.049**
Mumps	0.66 (0.44–1.00)	0.052	**0.39 (0.32–0.47)**	**<0.001**	**0.47 (0.40–0.55)**	**<0.001**	**0.61 (0.42–0.89)**	**0.011**	**0.35 (0.28–0.43)**	**<0.001**	**0.47 (0.39–0.56)**	**<0.001**
Pertussis	**0.30 (0.15–0.62)**	**0.002**	**0.04 (0.02–0.06)**	**<0.001**	**0.10 (0.06–0.15)**	**<0.001**	**0.21 (0.11–0.41)**	**<0.001**	**0.03 (0.01–0.05)**	**<0.001**	**0.11 (0.07–0.18)**	**<0.001**
Rubella	0.02 (0.001–1.12)	0.061	**0.01 (0.002–0.18)**	**0.002**	**0.01 (0.0004–0.23)**	**0.006**	**0.03 (0.001–0.81)**	**0.040**	**0.01 (0.0004–0.28)**	**0.008**	**0.08 (0.02–0.39)**	**0.002**
Scarlet fever	**0.27 (0.13–0.59)**	**0.001**	**0.22 (0.15–0.32)**	**<0.001**	**0.26 (0.19–0.35)**	**<0.001**	**0.18 (0.07–0.43)**	**<0.001**	**0.06 (0.04–0.11)**	**<0.001**	**0.16 (0.11–0.22)**	**<0.001**
Seasonal influenza	0.50 (0.07–3.52)	0.491	**0.08 (0.02–0.32)**	**<0.001**	**0.02 (0.01–0.08)**	**<0.001**	0.32 (0.06–1.84)	0.207	**0.12 (0.03–0.48)**	**0.004**	**0.03 (0.01–0.10)**	**<0.001**
Tuberculosis	0.88 (0.78–1.01)	0.071	1.02 (0.95–1.09)	0.642	0.97 (0.90–1.03)	0.286	0.83 (0.67–1.02)	0.082	0.9 (0.80–1.00)	0.062	**0.81 (0.72–0.91)**	**<0.001**
**Gastrointestinal and enteroviral disease**												
AHC	0.83 (0.38–1.80)	0.631	**0.48 (0.32–0.71)**	**<0.001**	**0.44 (0.30–0.64)**	**<0.001**	**1.79 (1.38–2.31)**	**<0.001**	**1.23 (1.07–1.40)**	**0.004**	**1.40 (1.24–1.59)**	**<0.001**
Bacterial dysentery	**0.66 (0.53–0.84)**	**<0.001**	**0.84 (0.75–0.93)**	**0.002**	**0.86 (0.77–0.96)**	**0.007**	**0.65 (0.51–0.82)**	**<0.001**	**0.78 (0.71–0.86)**	**<0.001**	**0.90 (0.81–0.99)**	**0.039**
Hepatitis A	1.04 (0.83–1.31)	0.744	1.00 (0.89–1.12)	0.991	0.95 (0.85–1.06)	0.337	1.26 (0.85–1.86)	0.257	**0.67 (0.53–0.86)**	**0.002**	**0.55 (0.42–0.71)**	**<0.001**
Hepatitis E	0.93 (0.73–1.17)	0.523	1.00 (0.88–1.14)	0.967	**0.86 (0.76–0.97)**	**0.014**	1.01 (0.82–1.24)	0.931	0.92 (0.82–1.03)	0.141	**0.89 (0.80–1.00)**	**0.044**
HFMD	0.04 (0.001–1.11)	0.062	**0.09 (0.04–0.19)**	**<0.001**	0.92 (0.61–1.39)	0.690	0.14 (0.002–8.96)	0.355	**0.13 (0.05–0.30)**	**<0.001**	0.81 (0.49–1.32)	0.393
Infectious diarrhea	0.59 (0.34–1.01)	0.059	1.05 (0.81–1.36)	0.730	0.84 (0.66–1.07)	0.162	0.98 (0.73–1.31)	0.900	0.92 (0.83–1.03)	0.162	**0.83 (0.72–0.95)**	**0.010**
Typhoid and paratyphoid	0.77 (0.56–1.05)	0.099	**0.79 (0.70–0.90)**	**<0.001**	**0.75 (0.66–0.85)**	**<0.001**	0.65 (0.35–1.19)	0.165	**0.77 (0.61–0.98)**	**0.039**	**0.78 (0.61–0.99)**	**0.049**
Amoebic dysentery	0.84 (0.47–1.51)	0.563	1.27 (0.97–1.68)	0.091	1.05 (0.79–1.39)	0.757	–	–	–	–	–	–
**Sexually transmitted or bloodborne diseases**												
Gonorrhea	0.96 (0.79–1.18)	0.722	**1.22 (1.12–1.34)**	**<0.001**	**1.18 (1.08–1.27)**	**<0.001**	**0.63 (0.52–0.76)**	**<0.001**	0.93 (0.85–1.01)	0.088	1.00 (0.92–1.08)	0.970
Syphilis	1.07 (0.91–1.25)	0.414	0.98 (0.92–1.04)	0.460	**0.85 (0.80–0.90)**	**<0.001**	1.02 (0.85–1.24)	0.809	0.98 (0.89–1.08)	0.674	0.91 (0.83–0.99)	0.034
HIV/AIDS	**1.71 (1.29–2.26)**	**<0.001**	**1.17 (1.02–1.34)**	**0.028**	0.91 (0.80–1.04)	0.173	1.06 (0.77–1.47)	0.721	**1.20 (1.03–1.40)**	**0.023**	1.02 (0.89–1.19)	0.746
Hepatitis B	**0.80 (0.67–0.95)**	**0.011**	1.00 (0.92–1.10)	0.931	0.95 (0.88–1.04)	0.266	**0.81 (0.67–0.97)**	**0.026**	0.98 (0.89–1.07)	0.627	**0.88 (0.80–0.96)**	**0.007**
Hepatitis C	**0.83 (0.76–0.91)**	**<0.001**	1.05 (1.00–1.10)	0.035	0.97 (0.92–1.03)	0.365	0.85 (0.69–1.05)	0.145	1.00 (0.90–1.12)	0.955	**0.90 (0.81–1.00)**	**0.045**
**Vector borne or zoonotic disease**												
Brucellosis	0.92 (0.65–1.30)	0.643	**1.33 (1.11–1.59)**	**0.003**	**1.67 (1.40–1.99)**	**<0.001**	1.13 (0.87–1.48)	0.355	**1.16 (1.02–1.33)**	**0.030**	**1.30 (1.13–1.49)**	**<0.001**
HFRS	1.05 (0.71–1.56)	0.809	0.99 (0.80–1.24)	0.946	**0.76 (0.62–0.93)**	**0.009**	0.74 (0.32–1.73)	0.494	**0.64 (0.43–0.96)**	**0.036**	1.10 (0.87–1.40)	0.431
Malaria	**0.26 (0.18–0.38)**	**<0.001**	**0.24 (0.19–0.31)**	**<0.001**	**0.41 (0.31–0.55)**	**<0.001**	**0.29 (0.17–0.50)**	**<0.001**	**0.09 (0.06–0.14)**	**<0.001**	**0.20 (0.14–0.29)**	**<0.001**

Hydatid disease	1.54 (0.50–4.72)	0.455	**3.15 (1.87–5.32)**	**<0.001**	**1.94 (1.18–3.19)**	**0.011**	1.15 (0.67–1.96)	0.610	1.18 (0.90–1.55)	0.225	1.21 (0.94–1.56)	0.144
Dengue	0.78 (0.001–916.71)	0.944	**0.004 (0.0001–0.18)**	**0.006**	**0.004 (0.001–0.02)**	**<0.001**	–	–	–	–	–	–
Typhus	0.57 (0.3–1.06)	0.080	0.91 (0.69–1.2)	0.521	0.91 (0.71–1.16)	0.439	–	–	–	–	–	–

* Data of Hubei and Tibet (both in South China) were not included in modelling due to unavailable healthcare visits data during the COVID-19 pandemic.
† HFMD, hand, foot and mouth disease; AHC, acute hemorrhagic conjunctivitis; HFRS, hemorrhagic fever with renal syndrome.
‡ Phase II: Feb 2020 and Mar 2020; Phase III: Apr 2020 to Aug 2020; Phase IV: Sep 2020 to Dec 2020. The reference period is Jan 2014 to Jan 2020.
-: GLM was not fitted for disease with median monthly case number is less than 50.
Statistically significant reductions (IRR<1) are displayed in bolded font.

Supplementary Table 3. Abbreviations and vaccine availability for the 31 notifiable infectious diseases included in this study.

	Disease	Abbreviation	Available vaccine*
**Respiratory diseases**			
1	Tuberculosis	TB	PIV
2	Scarlet fever	SF	-
3	Mumps	Mumps	PIV
4	Measles	Measles	PIV
5	Rubella	Rubella	PIV
6	Pertussis	Pertussis	PIV
7	Seasonal influenza	Flu	UIV
**Gastrointestinal or enteroviral diseases**			
8	Hepatitis A	Hepatitis A	PIV
9	Hepatitis E	Hepatitis E	UIV
10	Hand, foot and mouth disease	HFMD	UIV
11	Typhoid or paratyphoid	T/P	UIV
12	Bacterial dysentery	BD	-
13	Acute hemorrhagic conjunctivitis	AHC	-
14	Infectious diarrhea diseases	ID	-
15	Amoebic dysentery	AD	-
**Sexually transmitted or bloodborne diseases**			
16	Gonorrhea	Gonorrhea	-
17	Syphilis	Syphilis	-
18	HIV/AIDS	HIV/AIDS	-
19	Hepatitis B	Hepatitis B	PIV
20	Hepatitis C	Hepatitis C	-
**Vector-borne or zoonotic diseases**			
21	Dengue	Dengue	-
22	Malaria	Malaria	-
23	Japanese encephalitis	JE	PIV
24	Schistosomiasis	Schistosomiasis	-
25	Typhus	Typhus	UIV
26	Brucellosis	Brucellosis	-
27	Hemorrhagic fever with renal syndrome	HFRS	UIV
28	Anthrax	Anthrax	UIV
29	Rabies	Rabies	UIV
30	Hydatid disease	HD	-
31	Leptospirosis	Leptospirosis	-

* Vaccines for human use. PIV: Planned immunization vaccine. UIV: Unplanned immunization vaccines. -: No vaccines available.

Supplementary Figure 22. The weekly numbers of reported COVID-19 cases across the four intervention phases during 2020 in China. The four phases are defined according to the timeline of major intervention events: Jan 1 to Jan 22 (Phase I), Jan 23 to Apr 7 (Phase II), Apr 8 to Aug 31 (Phase III) and Sep 1 to Dec 31 (Phase IV). Jan 23 and Apr 7 mark the initiation and lifting of the lockdown of Wuhan, respectively, and Sep 1 marks the reopening of schools nationwide.

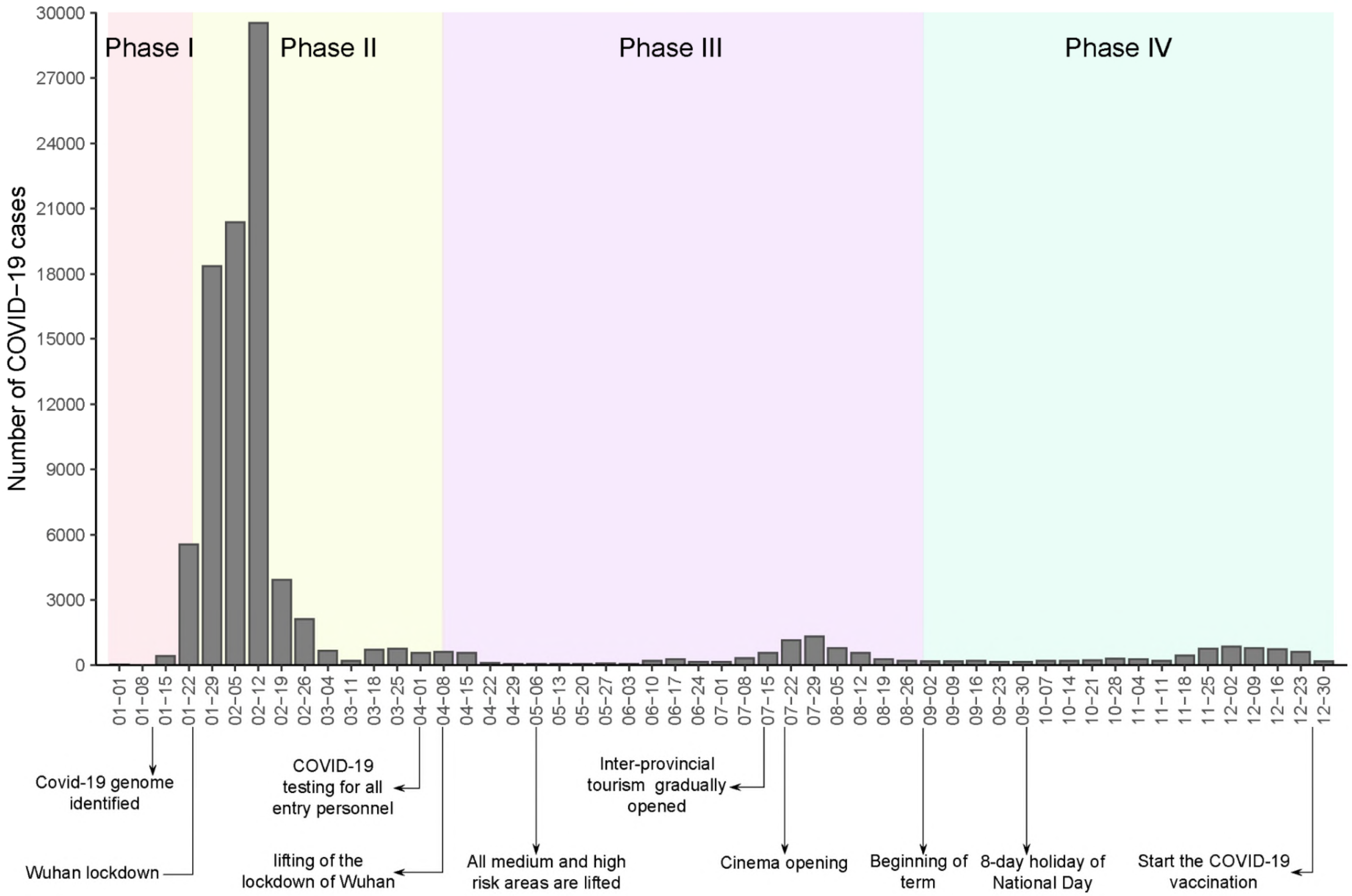

Supplementary Table 4. QAIC values of different choices for the functional form of the long-term year effect.

	South			North		
	**Flat**	**Linear**	**Quadratic**	**Flat**	**Linear**	**Quadratic**
**Respiratory diseases**						
Measles	12449.2	2556.0	2604.4	31323.6	16455.8	14026.4
Mumps	25446.8	15535.2	15884.2	14561.1	11875.3	11638.2
Pertussis	12834.1	5142.5	5167.6	7319.3	4635.6	4488.8
Rubella	33926.6	33363.2	14588.2	10118.1	10139.2	5321.8
Scarlet fever	4869.9	4580.0	4443.7	6558.3	6367.5	6481.1
Seasonal influenza	2567571.1	1283755.4	1048382.9	565177.1	314688.3	229892.5
Tuberculosis	8810.4	7862.2	7627.8	14474.8	12714.6	12166.2
**Gastrointestinal or enteroviral diseases**						
AHC						
