# Supplementary material for: Changes in notifiable infectious disease incidence in China during the COVID-19 pandemic
Source: Nat Commun. 2021 Nov 26;12:6923. doi: 10.1038/s41467-021-27292-7 (PMC8626444; doi:10.1038/s41467-021-27292-7)
Supplement: Supplementary file 2 — Description of Additional Supplementary Files [file 41467_2021_27292_MOESM2_ESM.pdf]

File Name: Supplementary Data 1
Description: Code and data for analyses
