# Supplementary material for: Changes in notifiable infectious disease incidence in China during the COVID-19 pandemic
Source: Nat Commun. 2021 Nov 26;12:6923. doi: 10.1038/s41467-021-27292-7 (PMC8626444; doi:10.1038/s41467-021-27292-7)
Supplement: Supplementary file 4 — Reporting Summary [file 41467_2021_27292_MOESM4_ESM.pdf]

# nature portfolio

Corresponding author(s): Li-Ping Wang, Yang Yang, Li-Qun Fang, Wei Liu

Last updated by author(s): Nov 2, 2021

# Reporting Summary

Nature Portfolio wishes to improve the reproducibility of the work that we publish. This form provides structure for consistency and transparency in reporting. For further information on Nature Portfolio policies, see our Editorial Policies and the Editorial Policy Checklist.

## Statistics

For all statistical analyses, confirm that the following items are present in the figure legend, table legend, main text, or Methods section.

| n/a | Confirmed | |
|---|---|---|
| ☐ | ☒ | The exact sample size (*n*) for each experimental group/condition, given as a discrete number and unit of measurement |
| ☒ | ☐ | A statement on whether measurements were taken from distinct samples or whether the same sample was measured repeatedly |
| ☐ | ☒ | The statistical test(s) used AND whether they are one- or two-sided<br>*Only common tests should be described solely by name; describe more complex techniques in the Methods section.* |
| ☐ | ☒ | A description of all covariates tested |
| ☒ | ☐ | A description of any assumptions or corrections, such as tests of normality and adjustment for multiple comparisons |
| ☐ | ☒ | A full description of the statistical parameters including central tendency (e.g. means) or other basic estimates (e.g. regression coefficient) AND variation (e.g. standard deviation) or associated estimates of uncertainty (e.g. confidence intervals) |
| ☐ | ☒ | For null hypothesis testing, the test statistic (e.g. *F*, *t*, *r*) with confidence intervals, effect sizes, degrees of freedom and *P* value noted<br>*Give P values as exact values whenever suitable.* |
| ☒ | ☐ | For Bayesian analysis, information on the choice of priors and Markov chain Monte Carlo settings |
| ☒ | ☐ | For hierarchical and complex designs, identification of the appropriate level for tests and full reporting of outcomes |
| ☐ | ☒ | Estimates of effect sizes (e.g. Cohen's *d*, Pearson's *r*), indicating how they were calculated |

*Our web collection on statistics for biologists contains articles on many of the points above.*

## Software and code

Policy information about availability of computer code

| | |
|---|---|
| Data collection | No software was used in the data collection. |
| Data analysis | R statistical software (version 4.0.3, R Development Core Team 2020) and R packages "tidyverse", "seasonal","tsModel", "forecast", "MASS", "cowplot", "patchwork", "conflicted" were used in the data analysis. No custom code was used in this analyses. |

For manuscripts utilizing custom algorithms or software that are central to the research but not yet described in published literature, software must be made available to editors and reviewers. We strongly encourage code deposition in a community repository (e.g. GitHub). See the Nature Portfolio guidelines for submitting code & software for further information.

## Data

Policy information about availability of data

All manuscripts must include a data availability statement. This statement should provide the following information, where applicable:

- Accession codes, unique identifiers, or web links for publicly available datasets
- A description of any restrictions on data availability
- For clinical datasets or third party data, please ensure that the statement adheres to our policy

Relevant data that support the findings of this study and model results generated as part of this study are publicly available within the paper and its Supplementary Information Files. Raw data are not publicly available and are protected due to data privacy laws, which were used under license for the current study, but are available upon reasonable request to the corresponding author and with permission from the data provider (Li-Ping Wang). The request will be responded within one week.

# Field-specific reporting

Please select the one below that is the best fit for your research. If you are not sure, read the appropriate sections before making your selection.

- [x] Life sciences
- [ ] Behavioural & social sciences
- [ ] Ecological, evolutionary & environmental sciences

For a reference copy of the document with all sections, see nature.com/documents/nr-reporting-summary-flat.pdf

# Life sciences study design

All studies must disclose on these points even when the disclosure is negative.

| | |
|---|---|
| Sample size | From 2014 to 2020, all of the individuals (over 53 million individuals) who were diagnosed as 31 selected notifiable infectious diseases were all extracted from the China Information System for Disease Control and Prevention (CISDCP) and were included in the analysis. |
| Data exclusions | Diseases with less than 2000 reported cases during this period (filaria, hepatitis D, diphtheria, neonatal tetanus, meningococcal meningitis, cholera, plague, kala-azar, poliomyelitis, avian influenza H7N9, H5N1, H1N1 and SARS) and leprosy (direct contact transmission) were excluded. |
| Replication | Reproducibility of experimental findings was not applicable because our study is observational. |
| Randomization | Randomization used to allocate participates into experimental groups was not applicable because our study is observational. |
| Blinding | Blinding was not applicable because our study is observational. |

# Reporting for specific materials, systems and methods

We require information from authors about some types of materials, experimental systems and methods used in many studies. Here, indicate whether each material, system or method listed is relevant to your study. If you are not sure if a list item applies to your research, read the appropriate section before selecting a response.

## Materials & experimental systems

| n/a | Involved in the study | |
|---|---|---|
| x | | Antibodies |
| x | | Eukaryotic cell lines |
| x | | Palaeontology and archaeology |
| x | | Animals and other organisms |
| | x | Human research participants |
| x | | Clinical data |
| x | | Dual use research of concern |

## Methods

| n/a | Involved in the study | |
|---|---|---|
| x | | ChIP-seq |
| x | | Flow cytometry |
| x | | MRI-based neuroimaging |

## Human research participants

Policy information about studies involving human research participants

| | |
|---|---|
| Population characteristics | From 2014 to 2020, the 31 selected notifiable infectious diseases were diagnosed in over 53 million individuals with a median age of 17 (IQR:2–46) years, 59.83% of whom were male. |
| Recruitment | All of the cases who were diagnosed as the 31 selected notifiable infectious diseases during the study period were extracted from the China Information System for Disease Control and Prevention (CISDCP) and included in the current analysis. Only confirmed cases were included and the the suspected cases were excluded. All of the diseases were diagnosed according to their standard diagnostic criteria. Thus, there was no sampling or self-report bias in the current study. |
| Ethics oversight | It was determined by the National Health Commission of China that the collection of data on notifiable infectious diseases was part of continuing public health surveillance of infectious diseases and was exempt from institutional review board assessment. All the data of cases used in this study were anonymized and personal identification information (e.g., name and street address) is not included in the data, and this study was approved by Chinese Center for Disease Control and Prevention (202026). |

Note that full information on the approval of the study protocol must also be provided in the manuscript.
